# Mining for genes related to pistil abortion in *Prunus sibirica* L.

**DOI:** 10.7717/peerj.14366

**Published:** 2022-11-15

**Authors:** Jianhua Chen, Jian Zhang, Quangang Liu, Xinxin Wang, Jiaxing Wen, Yongqiang Sun, Shengjun Dong

**Affiliations:** College of Forestry, Shenyang Agricultural University, Shenyang, China

**Keywords:** *Prunus sibirica*, Pistil abortion, Transcriptome sequencing, Differentially expressed genes (DEGs), qRT-PCR

## Abstract

In *Prunus sibirica*, the phenomenon of pistil abortion is very common and seriously affects its fruit quality and yield; however, the molecular mechanisms of pistil abortion remains unclear. In this study, we identified differentially expressed genes (DEGs) and pathways associated with pistil abortion using transcriptome sequencing. After comparative analysis, a total of 1,950 DEGs were identified, of which 1,000 were upregulated, and 950 were downregulated. Gene Ontology (GO) functional enrichment analysis of DEGs showed that metabolic process, cellular process, single-organism process, membrane, membrane part, cell, binding, catalytic activity, and transporter activity contained the largest number of DEGs. Kyoto Encyclopedia of Genes and Genomes (KEGG) pathway enrichment analysis showed that the plant-pathogen interaction, starch and sucrose metabolism, and plant hormone signal transduction pathways contained the largest number of DEGs. The NAC, bHLH, and B3 transcription factor families contained the largest number of DEGs. qRT-PCR detection confirmed that the gene expression levels were consistent with the transcriptome sequencing results. This study provides a theoretical basis and scientific basis for further research on the molecular mechanisms of *P. sibirica* pistil abortion.

## Introduction

*Prunus sibirica* is a deciduous fruit species belonging to the Rosaceae family (Wang & Yu, 2012), and is mostly distributed in regions in eastern Siberia, eastern and southeastern Mongolia, far east Russia, and north and northeastern China (Niu et al., 2014). *P. sibirica* is a multipurpose tree species with ecological and economic value. This species has a strong resistance to cold, drought, and barren conditions, and is well-adapted to various types of environments (Wang et al., 2014). Its well-developed roots play an important role in wind prevention, sand fixation, and soil and water conservation (Mai et al., 2020). *P. sibirica* is widely used in various fields such as food, medicine, and industry. Almonds can be consumed on their own or as part of several food products in the form of nutlets, almond oil, and almond milk (Wang et al., 2014). Almonds contain proteins, lipids, carbohydrates, dietary fibre, micronutrients, and phytochemicals (Grundy, Lapsley & Ellis, 2016). Their oil content is as high as 50%, of which approximately 95% are unsaturated fatty acids (FAs) (Mai et al., 2020), which have positive health benefits in heart disease, diabetes, and obesity (Grundy, Lapsley & Ellis, 2016). Therefore, the yield of *P. sibirica* is important to farmers’ income of in *P. sibirica-* growing areas.

Pistil abortion is a ubiquitous and diverse phenomenon with a complex mechanism. This phenomenon has been observed in many plants, such as *Olea europaea* (Rosatia et al., 2011), *Punica granatum* (Chen et al., 2017a), and *Prunus mume* (Shi et al., 2020). According to the pistil development process and the structural characteristics of abortion, pistil abortion can be divided into four types: stagnation or retardation of the pistil differentiation process (Shi et al., 2011), abnormal integument development (Chen et al., 2017a), abnormal style structure (Wetzstein et al., 2011; Zhang et al., 2013; Sun, 2014) and arrested embryo sac development (Zhang, 2017). Whether the fruit tree can blossom and bear fruit normally depending on whether the pistil develops normally.

Pistil abortion is also common in *P. sibirica* (Yin et al., 2018). The phenomenon of flower falling is critical, resulting in the yield decline and seriously restricting its industry development. At present, studies on pistil abortion in *Prunus* species have mainly focused on period, type, and reason for abortion. Wang et al. (2000) showed that the pistil abortion period began in late February, and the peak pistil abortion period was from the big balloon to the full-flowering period. Li & Ma (2001) observed that different ovary malformations occur easily. Shen et al. (2007) believed that there were many reasons for pistil abortion, and we should focus on the abortion caused by internal reasons. *Prunus armeniaca* var. *glabra* is often aborted due to adverse environmental conditions such as low temperatures in early spring, resulting in shorter styles and pistil degeneration (Chen et al., 2021). Our previous studies found that No. 28 was the typical *P. sibirica* clone with normal pistils and No. 595 was the typical *P. sibirica* clone with abortive pistils. No. 28 clone developed completely, and can pollinate and bear fruits as well. The pistil abortion rate of No. 595 clone was 95.09%, and its pistil abortion type was abnormal style structure. The pistil abortion occurs in the dew white stage, pistil exhibited degeneration and dissolution, and completely disintegrated and disappeared at the full flowering stage. From the dormancy release to the full flowering stage, the soluble protein and soluble sugar of *P. sibirica* flower buds showed a “rising-declining-rising” trend, starch showed a continuous declining trend, and total sugar showed a “declining-rising” trend. At the key stage of pistil abortion (dew white stage, March 27, 2021), the contents of soluble protein, soluble sugar and total sugar in abortive flowers were extremely significantly lower than those in normal flowers. The pistil abortion of *P. sibirica* were mainly related to the insufficient supply of soluble protein, soluble sugar and total sugar in flower buds (Zhang, 2022).

The transcriptome, a bridge between the genome and proteome, plays an important role in transmitting genetic information and performing biological functions. In recent years, with the reduced sequencing cost and improved technology, transcriptome sequencing technology has been widely used to study plant pistil abortion. Transcriptome sequencing technology has been previously applied to study the pistil abortion of *Prunus mume* (Shi et al., 2020), *Punica granatum* (Chen et al., 2017a), pumpkin (Li et al., 2020), and *Prunus armeniaca* var. *glabra* (Zhao, 2020). The potential functional genes screened out by transcriptome sequencing provide a theoretical basis for subsequent research on the biological gene functions of genes through reverse genetics, and the process was time- and cost-efficient. The key genes and related molecular mechanisms regulating pistil abortion of *P. sibirica* have not been reported. Therefore, from the genetic perspective, this study screened the differentially expressed candidate genes by transcriptome sequencing of flower bud samples during the critical abortion period. First, we performed bioinformatics analysis on the DEGs related to pistil abortion in *P. sibirica* to provide supportive data and a theoretical basis for further studies. Next, we explored the DEGs related to pistil abortion in *P. sibirica* through transcriptome sequencing to provide a scientific basis for molecular-assisted *P. sibirica* breeding, overcoming pistil abortion during cultivation, and improving fruit yield and quality.

## Materials and Methods

### Plant materials

Seven-year-old *P. sibirica* clones in the National Forest Germplasm Resource Preservation Repository for *Prunus* species of Shenyang Agricultural University (Kazuo, Liaoning, China) were selected as the experimental materials. They were grown with a row spacing of 3m and an in-row distance of 2m. Clone No. 595 (experimental group) was selected as the material with abortive pistils (APt), and clone No. 28 (control group) was selected as the material with normal pistils (NPt). Three plants with good and uniform growth conditions were selected from each clone, and flower bud samples of the white stage (about 30 buds per sample) were collected for transcriptome sequencing on March 27, 2021. There were no extreme climatic conditions such as low temperature, high temperature or flood in the test site. For the convenience of description, the sequencing results from three repeats each of *P. sibirica* with abortive pistils and normal pistils were denoted as APt1, APt2, APt3, and NPt1, NPt2, NPt3, respectively.

### Total RNA extraction and quality control

Total RNA was extracted from mixed flower buds samples from 3 plants using the EASYspin Plant microRNA Kit (Aidlab, China). RNA concentration and purity were measured using a NanoDrop 2000 (Thermo Fisher Scientific, Wilmington, DE, USA). RNA integrity was assessed using the RNA Nano 6000 Assay Kit with the Agilent Bioanalyzer 2100 system (Agilent Technologies, Santa Clara, CA, USA).

### Library preparation and transcriptome sequencing

After the samples were tested for their quality, library construction for sequencing was performed. The detailed process was as follows: First, mRNA was isolated from total RNA by Oligo(dT)-attached magnetic beads, and was randomly fragmented in the fragmentation buffer. Then, the fragmented mRNA and random hexamer primer were used to synthesize the first-strand cDNA. The second-strand cDNA was synthesized by adding buffer, dNTPs, RNase H, and DNA polymerase I. The cDNA was purified using AMPure XP beads. Next, the double-stranded cDNA was end-repaired and A-tailed for adapter-ligated. AMPure XP beads were used to select 300∼400 bp fragments. Finally, cDNA libraries were obtained by PCR enrichment.

Qubit 2.0 and Agilent 2100 systems were used to examine cDNA concentration and insert size to ensure the library quality. The high-quality libraries with concentration greater than 2 nM were obtained by qPCR. Three biological replicates were used for each group for sequencing. The constructed libraries were sequenced on an Illumina Novaseq 6000 platform, 150 bp paired-end reads were generated, and the amount of data was about 6G clean data for each sample. The statistical power analysis of this experimental design was calculated using the R package RNASeqPower (Table S1).

### Raw data quality control

Raw data (raw reads) of fastq format were first processed using in-house Perl scripts. In this step, clean data (clean reads) were obtained from the raw data by removing reads containing adapters, reads containing poly-N, and low-quality reads. At the same time, Q20, Q30, GC content and sequence duplication level of the clean data were calculated. All downstream analyses were based on high-quality clean data.

### Sequence alignment

Sequence alignment and follow-up analysis were conducted using the *P. sibirica* genome (https://www.rosaceae.org/Analysis/10254124) as a reference genome. Hisat2 tools soft (Kim, Langmead & Salzberg, 2015) were used to map the reference genome.

### Correlation assessment of replicates

Pearson’s correlation coefficient was used to evaluate the reproducibility of biological replicates (Liu et al., 2018). A closer R^2^ value of 1 indicates better reproducibility between the two samples.

### Gene functional annotation

In order to obtain the annotation information of unigenes, the unigene sequences were compared against the NCBI non-redundant protein sequences (Nr), NCBI non-redundant nucleotide sequences (Nt), protein family (Pfam), clusters of orthologous groups of proteins (KOG/COG), Swiss-Prot protein sequence database, KEGG ortholog (KO) and Gene Ontology (GO).

### Quantification of gene expression levels

Quantification of gene expression levels was estimated by fragments per kilobase of transcript per million fragments mapped.

### DEGs and transcription factor analysis

FPKM was applied to measure the expression level of a gene or transcript by StringTie using maximum flow algorithm (Florea, Song & Salzberg, 2013). Raw counts were input, and low expression was filtered. Differential expression analysis was performed using the edgeR (Robinson, McCarthy & Smyth, 2010). The analysis started by normalizing the input count. The false discovery rate (FDR) <0.01 & fold change ≥2 were set as the threshold for significantly differential expression. GO enrichment analysis of the DEGs was performed using the GOseq R packages based on Wallenius non-central hyper-geometric distribution (Young et al., 2010). KOBAS software (Mao et al., 2005) was used to test the statistical enrichment of DEGs in KEGG pathways. Transcription factor analysis was performed using iTAK 1.2 software. The genes sets of KEGG pathway and GO terms on molecular functions, biological processes and cellular components were employed in gene set enrichment analysis (GSEA). The log2FC of each differential group was used as the score of the background gene set to analyze the enrichment of the gene set. Enriched gene sets were identified as *p*-value<0.001 and FDR<0.05 (Khan et al., 2017).

### Validation of transcriptome sequencing results

Template cDNA was generated using PrimeScript™ RT Master Mix (Perfect Real Time) (RR036A; TaKaRa Bio, Shiga, Japan) for quantitative real-time PCR (qRT-PCR). The PCR reactions for reverse transcription (10 µL total volume) contained 5 × PrimeScript RT Master Mix (2 µL), RNase Free dH_2_O (7 µL), and total RNA (1 µL). The reaction conditions were 37 °C for 15 min, 85 °C for 5 s, and 4 °C for termination. Gene-specific primers (Table S2) were designed using Primer Premier 5.0. qRT-PCR was performed in three biological replicates. The gene primers used in the qRT-PCR experiments were listed in Table S2. 18SrRNA was used as a reference gene for qRT-PCR (Jin, 2018). qRT-PCR was performed using a StepOnePlus real-time PCR system. qRT-PCR was performed using TB Green^®^ Premix Ex Taq™ II (Tli RNaseH Plus) (TaKaRa Bio, Shiga, Japan). The PCR reactions (20 µL total volume) contained 10 µL 5 × TB Green Premix Ex Taq II (Tli RNaseH Plus), 6 µL RNase-free water, 0.8 µL upstream and downstream primers each, 2 µL cDNA template, 0.4 µL ROX reference Dye (50×). qRT-PCR was performed using three-step qPCR. The reaction conditions were 95 °C for 30 s, followed by 40 cycles of 95 °C for 5 s, 55 °C for 30 s, and 72 °C for 30 s.

## Results

### RNA isolation, library construction, and sequencing

RNA concentration of each sample ranged from 331.5 ng/ul to 925.2 ng/ul, OD260/280 ranged from 2.10 to 2.18, OD260/230 ranged from 1.16 to 2.21, RIN value ranged from 8.7 to 9.2, 28S/18S ranged from 1.94 to 2.09 (Table S3). The results showed that the quality of obtained total RNA was satisfying, and could meet the experimental requirements.

After quality control of sequencing data, 44.96 Gb clean data was obtained. Clean reads ranged from 22,462,256 to 27,048,846, clean bases ranged from 6,713,330,540 to 8,096,571,394, GC Content ranged from 45.89% to 46.03%, Q30 value ranged from 92.01% to 92.92% (Table S4). The results showed that the amount of data met the quality requirements for subsequent analysis.

### Power analysis

The mean power of all DEGs detected with FDR <0.01 and fold change ≥2 was 56.85%. The power value of 19.54% DEGs was above 60.00%, and the power value of 93.33% DEGs was above 50.00% (Table S1).

### Correlation assessment of biological replicates

**Figure 1:**
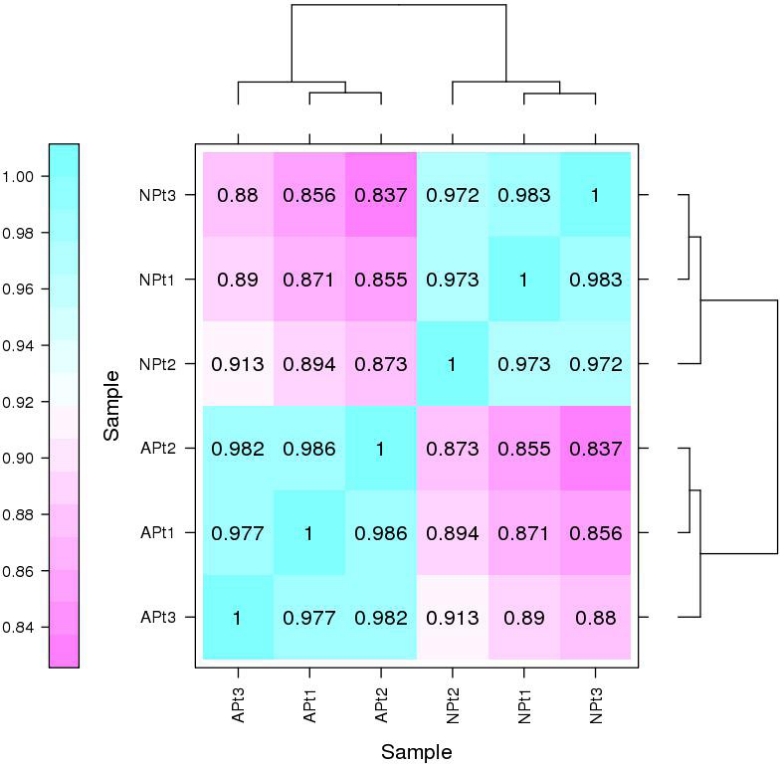
Correlation between sequencing samples in *P*. *sibirica*.

The Pearson correlation coefficients among the three biological replicates of each experimental group were 0.986 (APt1/APt2), 0.977 (APt1/APt3), and 0.982 (APt2/APt3). The Pearson correlation coefficients among the three biological replicates of each control group were 0.973 (NPt1/NPt2), 0.983 (NPt1/NPt3), and 0.972 (NPt2/NPt3) (Fig. 1). The correlations between the samples were all greater than 0.9, demonstrating an excellent internal consistency, which met the requirements for further biological analysis.

### The results of sequence alignment

Compared to the reference genome, the mapped rate of the experimental group samples ranged from 92.87% to 93.04%, and that of the control group samples ranged from 92.38% to 93.13%. The rate of reads map to ‘+’ and the rate of reads map to ‘−’ was similar (Table S5). The results showed that the quality and data volumes of transcriptome sequencing were relatively high, which met the requirements for subsequent data assembly and processing.

### Gene function annotation

A total of 30,627 unigenes were annotated in least one database, accounting for 89.9% of the total unigenes. The highest annotation rate was obtained in the NR database, which was assigned 30,543 (89.7%) unigenes. In the GO database, 23,405 (68.7%) unigenes were annotated. In the eggNOG database, 23,357 (68.6%) unigenes were annotated. In the Pfam database, 21,616 (63.5%) unigenes were annotated. In the KEGG database, 18,941 (55.6%) unigenes were annotated. In the Swiss-Prot database, 18,634 (54.7%) unigenes were annotated. In the KOG database, 13,897 (40.8%) unigenes were annotated. In the COG database, 8256 (24.2%) unigenes were annotated (Table S6).

### Differential gene expression analysis

**Figure 2:**
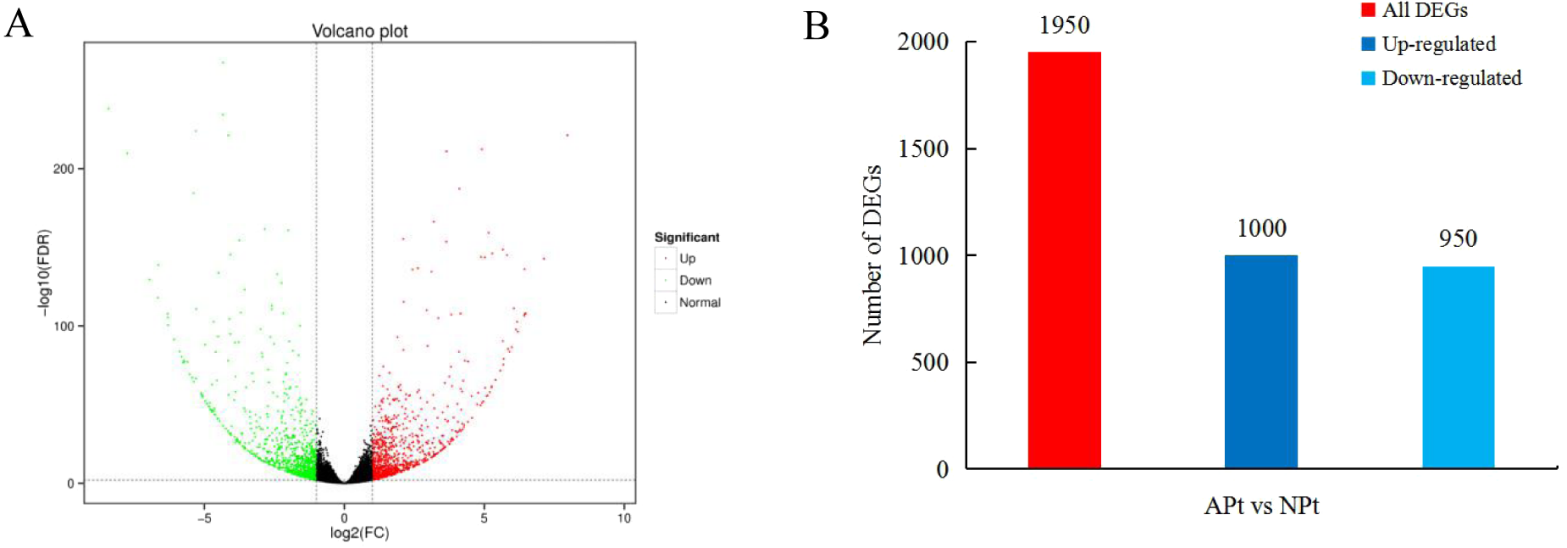
Distribution plots of DEGs in *P*. *sibirica*. (A) Volcano-plots, (B) Bar graphs.

A total of 1950 DEGs related to pistil abortion in *P. sibirica* were identified, including 1,000 upregulated genes and 950 downregulated genes (Fig. 2).

### GO enrichment analysis of DEGs

**Figure 3:**
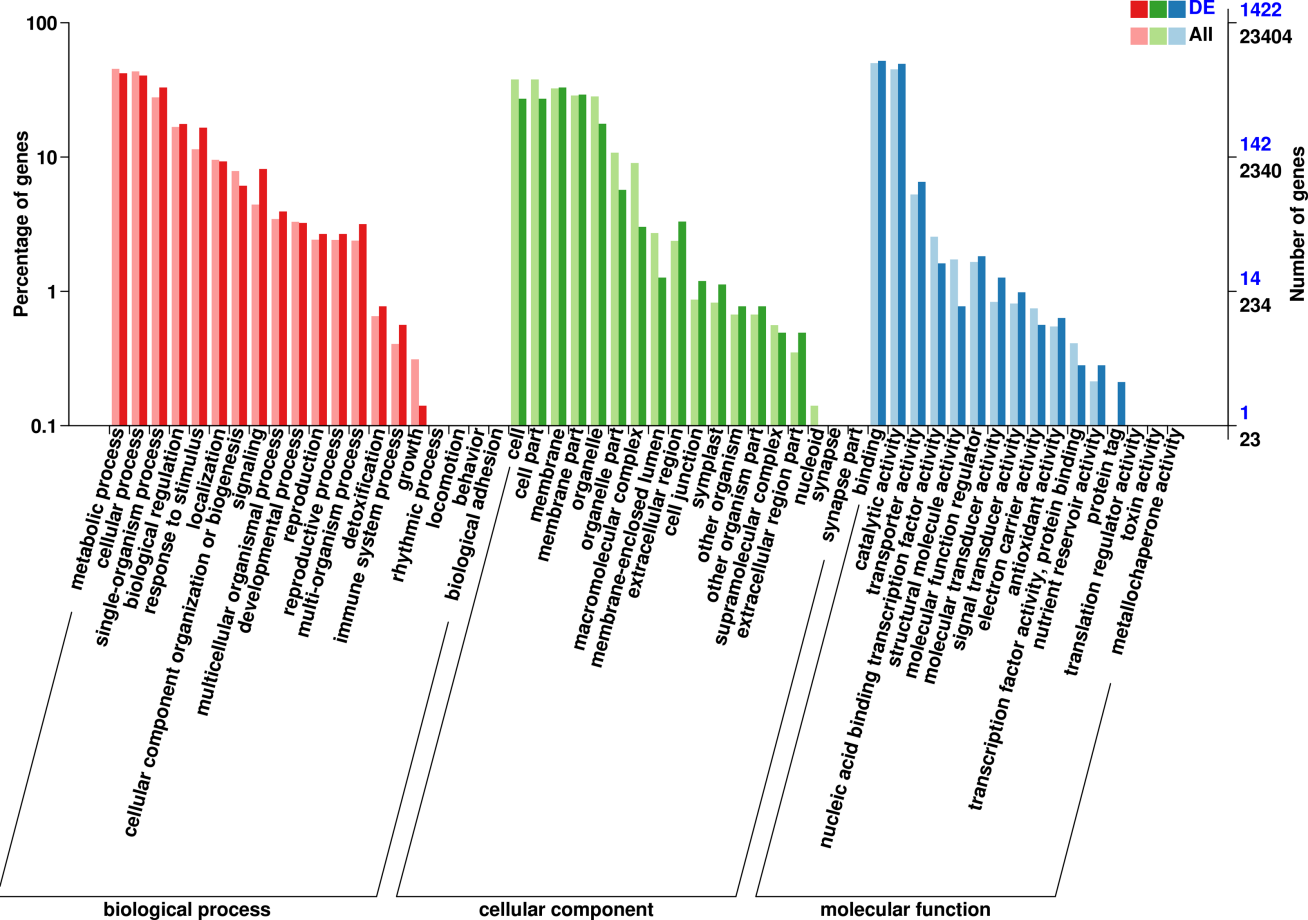
GO functional classification of DEGs in *P*. *sibirica*.

The 1,422 identified DEGs were annotated and were assigned, 20 terms belong to biological process, 18 terms belong to cellular component, and 16 terms belong to molecular function. Biological processes included predominantly metabolic process (597 genes), cellular process (575 genes), single-organism process (468 genes), biological regulation (251 genes), response to stimulus (235 genes), localization (132 genes), signaling (116 genes), cellular component organization or biogenesis (87 genes), multicellular organismal process (56 genes), developmental process (46 genes), multi-organism process (45 genes), reproduction (38 genes), and reproductive process (38 genes). Cellular component included predominantly membrane (469 genes), membrane part (415 genes), cell (386 genes), cell part (386 genes), organelle (252 genes), organelle part (81 genes), extracellular region (47 genes), extracellular region (43 genes), membrane-enclosed lumen (18 genes), cell junction (17 genes), and symplast (16 genes). Molecular function included predominantly binding (739 genes), catalytic activity (704 genes), transporter activity (93 genes), molecular function regulator (26 genes), nucleic acid binding transcription factor activity (23 genes), molecular transducer activity (18 genes), signal transducer activity (14 genes), and structural molecule activity (11 genes) (Fig. 3, Table S7).

### KEGG pathway enrichment analysis of DEGs

**Figure 4:**
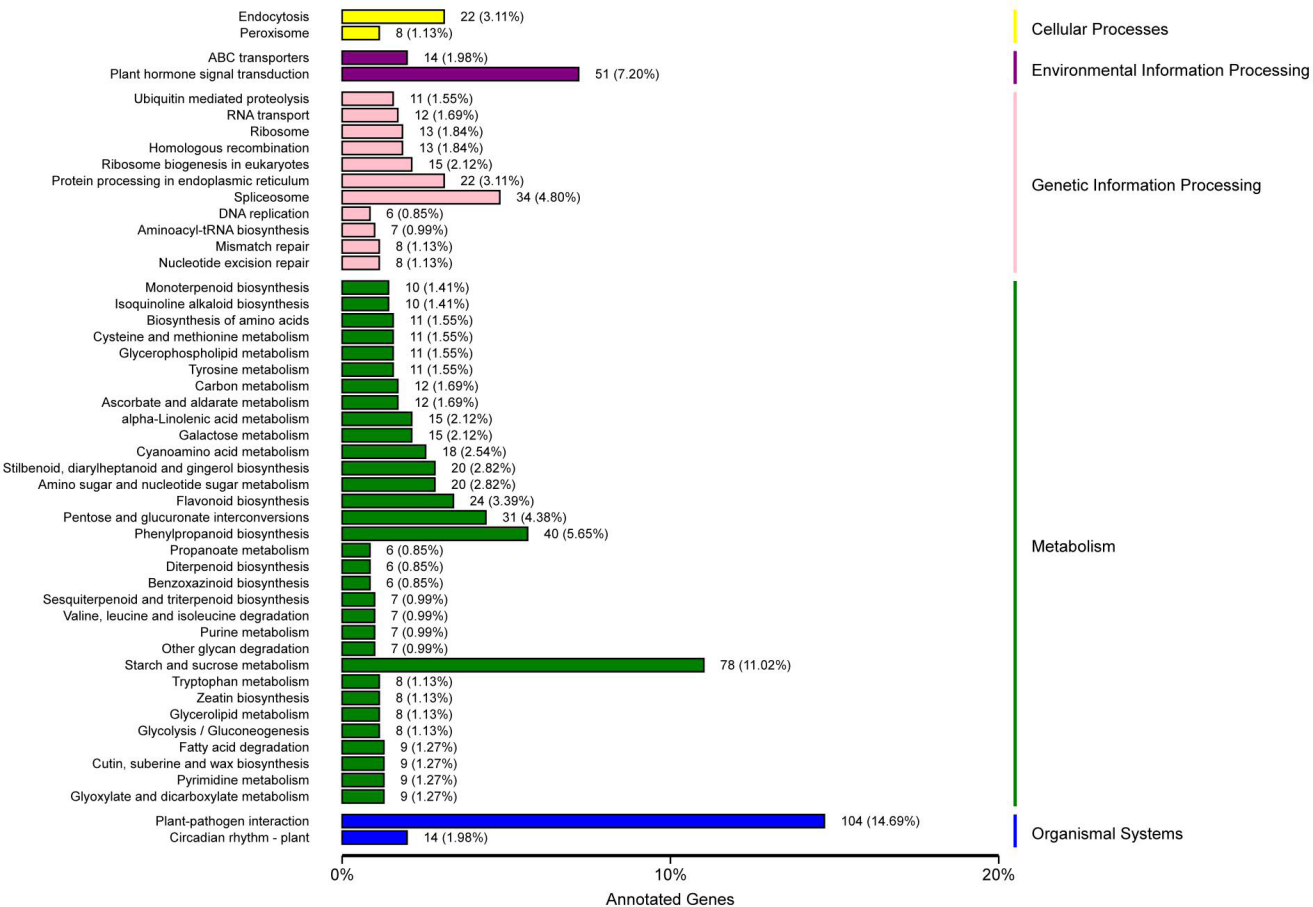
KEGG classification of DEGs in *P*. *sibirica*.

KEGG pathway analysis showed that 708 unigenes were annotated and assigned to 114 pathways. The 114 KEGG pathways were divided into five KEGG categories: metabolism, genetic information processing, organismal systems, environmental information processing, and cellular processes, including 401, 131, 118, 67, and 34 DEGs, respectively. In the metabolism pathway, there were additional genes involved in starch and sucrose metabolism (78 genes), phenylpropanoid biosynthesis (40 genes), and pentose and glucuronate interconversions (31 genes). In the genetic information processing pathway, additional genes were involved in spliceosome (34 genes), protein processing in endoplasmic reticulum (22 genes), and ribosome biogenesis in eukaryotes (15 genes). In the organismal systems pathway, the largest number of genes (104 genes) were involved in plant-pathogen interaction, and some (14 genes) were involved in the circadian rhythm-plant. In the environmental information processing pathway, the genes involved in plant hormone signal transduction were the highest (51 genes), followed by those involved in ABC transporters (14 genes). In the cellular processes pathway, the genes involved in endocytosis were the most prevalent (22 genes), followed by genes involved in peroxisome (eight genes) (Fig. 4, Table S8).

**Figure 5:**
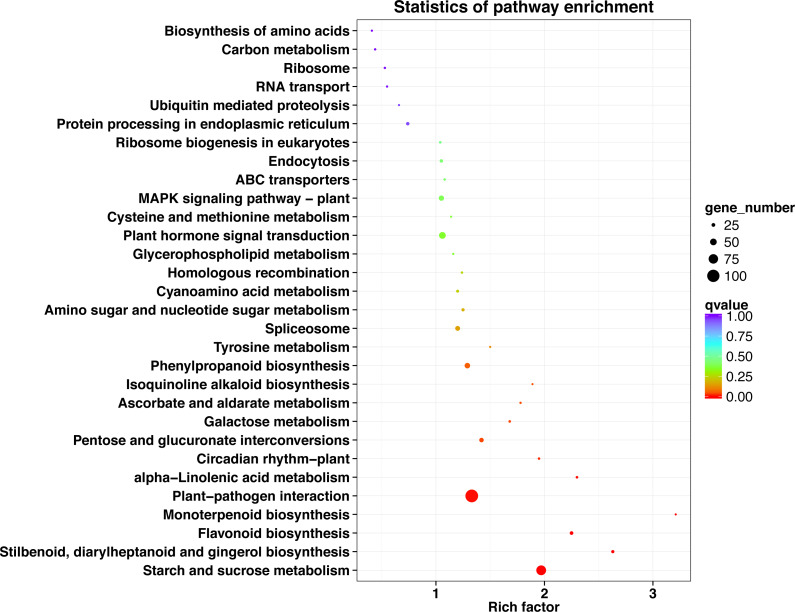
KEGG pathway enrichment of DEGs in *P*. *sibirica*.

The top 30 KEGG pathways with the most number of annotated genes were plant-pathogen interaction, starch and sucrose metabolism, plant hormone signal transduction, phenylpropanoid biosynthesis, MAPK signaling pathway-plant, spliceosome, pentose and glucuronate interconversions, flavonoid biosynthesis, protein processing in endoplasmic reticulum, endocytosis, stilbenoid, diarylheptanoid and gingerol biosynthesis, amino sugar and nucleotide sugar metabolism, cyanoamino acid metabolism, ribosome biogenesis in eukaryotes, alpha-Linolenic acid metabolism, galactose metabolism, circadian rhythm-plant, ABC transporters, ribosome, homologous recombination, ascorbate and aldarate metabolism, RNA transport, carbon metabolism, cysteine and methionine metabolism, glycerophospholipid metabolism, biosynthesis of amino acids, ubiquitin mediated proteolysis, tyrosine metabolism, isoquinoline alkaloid biosynthesis, and monoterpenoid biosynthesis (Fig. 5, Table S9).

### Clusters of orthologous genes (COG) annotation

**Figure 6:**
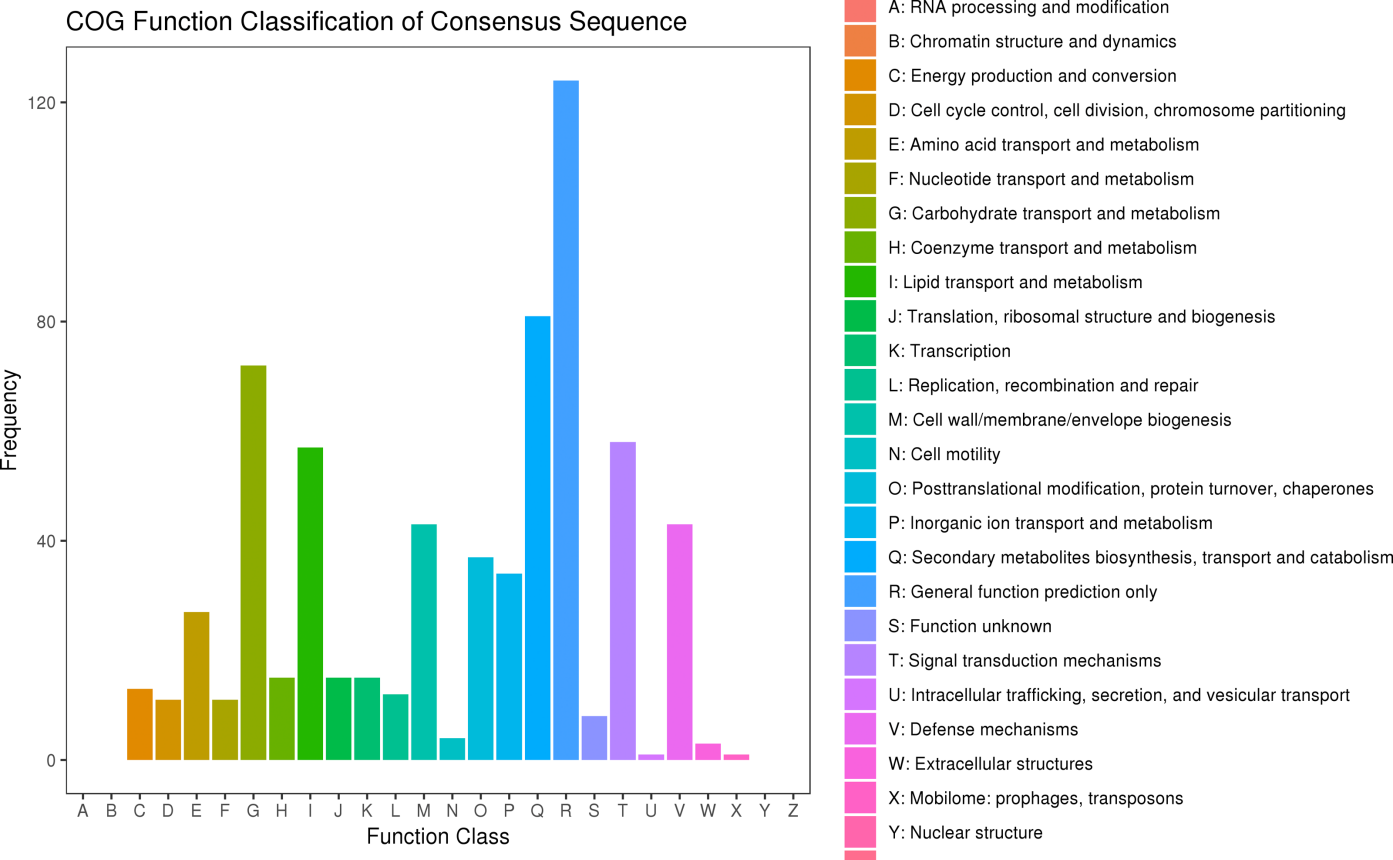
COG classification of DEGs in *P*. *sibirica*.

After searching the COG database, 577 unigenes were annotated to 22 COG categories. General function prediction only had the largest number of DEGs (124 genes). The second most prevalent DEGs were secondary metabolites biosynthesis, transport, and catabolism (81 DEGs), carbohydrate transport and metabolism (72 DEGs), signal transduction mechanisms (58 DEGs), and lipid transport and metabolism (57 DEGs) (Fig. 6, Table S10).

### Gene set enrichment analysis (GSEA)

**Figure 7:**
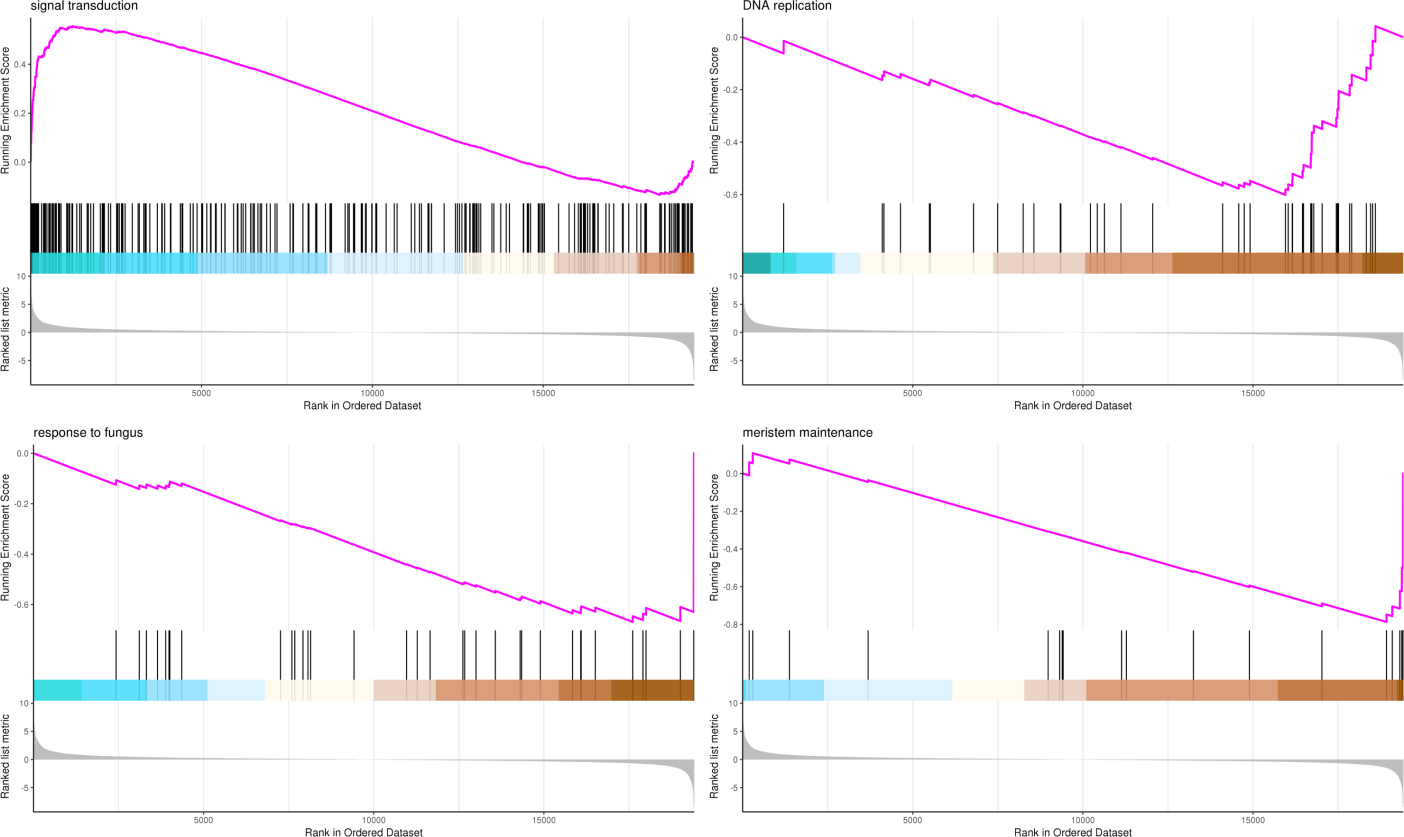
GSEA analysis based on biological process of GO functional classification in *P*. *sibirica*. Note: In the upper figure, *X*-axis was position of gene set after ordering, and *Y*-axis was enrichment score. The lines on the top represent genes in the gene set. Purple curve showed the enrichment score of each gene set across positions. In the lower figure, *X*-axis was position of gene set after ordering. *Y*-axis was score. Each line represents a gene in gene set. The length of lines indicates corresponding score.

**Figure 8:**
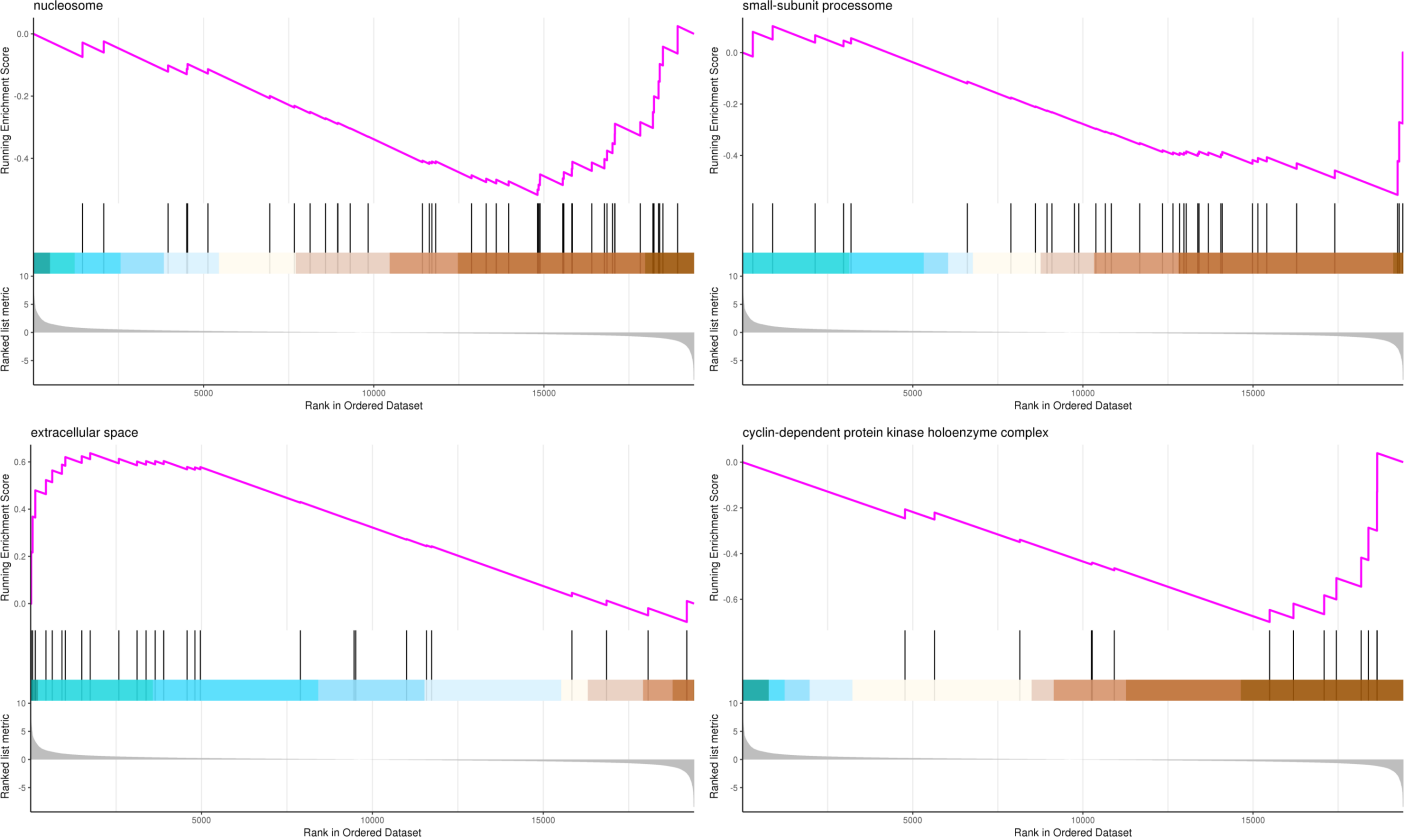
GSEA analysis based on cellular component of GO functional classification in *P*. *sibirica*. In the upper figure, X-axis was position of gene set after ordering, and Y -axis was enrichment score. The lines on the top represent genes in the gene set. Purple curve showed the enrichment score of each gene set across positions. In the lower figure, X-axis was position of gene set after ordering. Y -axis was score. Each line represents a gene in gene set. The length of lines indicates corresponding score.

**Figure 9:**
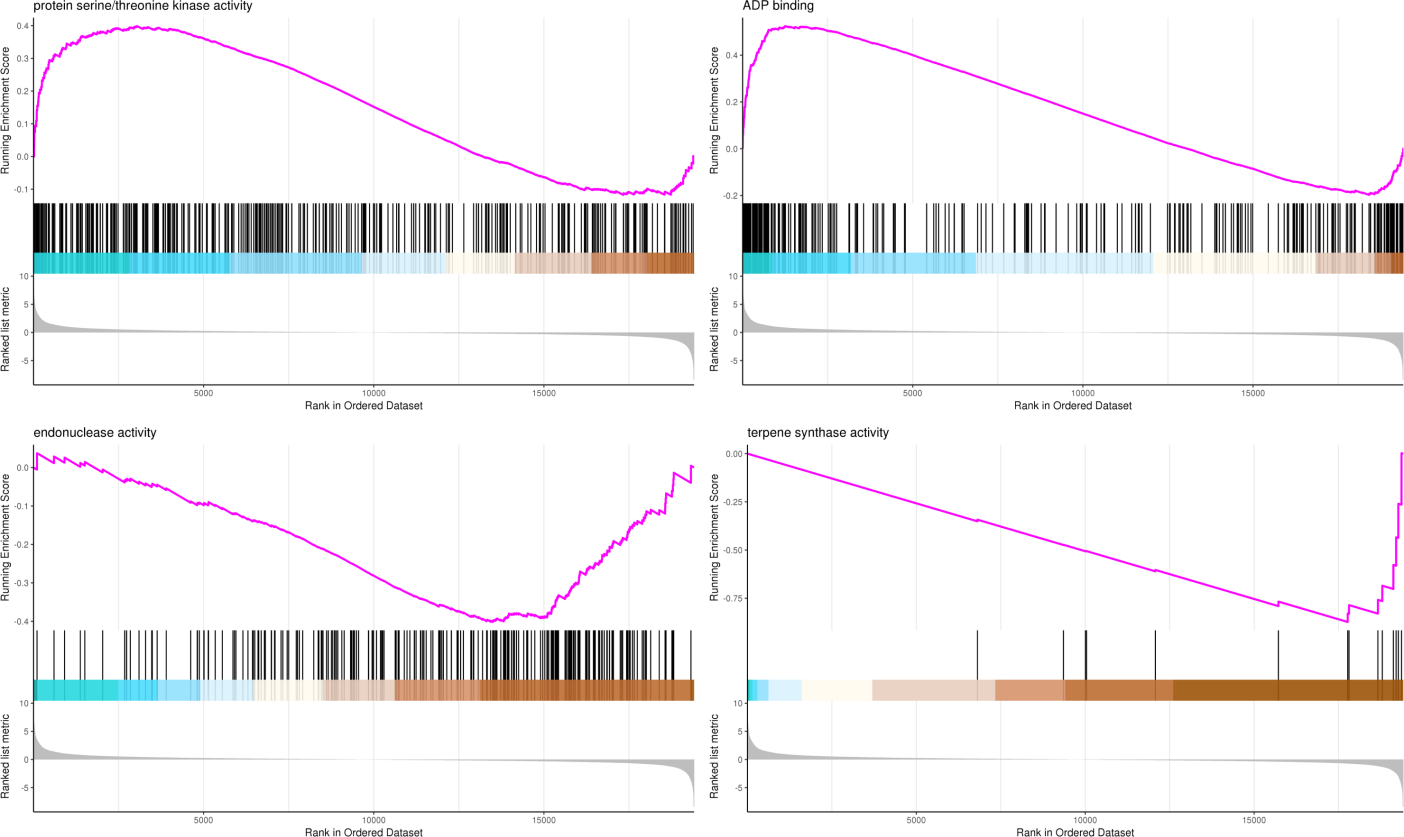
GSEA analysis based on molecular function of GO functional classification in *P*. *sibirica*. In the upper figure, X-axis was position of gene set after ordering, and Y -axis was enrichment score. The lines on the top represent genes in the gene set. Purple curve showed the enrichment score of each gene set across positions. In the lower figure, X-axis was position of gene set after ordering. Y -axis was score. Each line represents a gene in gene set. The length of lines indicates corresponding score.

The most significant four pathways of biological process in the GSEA-GO analysis were signal transduction (275 genes), DNA replication (45 genes), response to fungus (36 genes), and meristem maintenance (20 genes) (Fig. 7). The most significant four pathways of cellular component in the GSEA-GO analysis were nucleosome (43 genes), small-subunit processome (34 genes), extracellular space (27 genes), and cyclin-dependent protein kinase holoenzyme complex (14 genes) (Fig. 8). The most significant four pathways of molecular function in the GSEA-GO analysis were protein serine/threonine kinase activity (362 genes), ADP binding (308 genes), endonuclease activity (241 genes), and terpene synthase activity (14 genes) (Fig. 9, Table S11).

**Figure 10:**
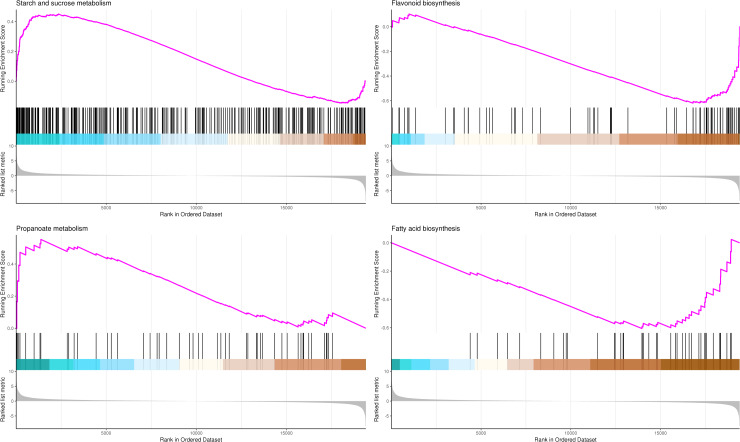
GSEA analysis based on KEGG pathway in *P*. *sibirica*. In the upper figure, X-axis was position of gene set after ordering, and Y -axis was enrichment score. The lines on the top represent genes in the gene set. Purple curve showed the enrichment score of each gene set across positions. In the lower figure, X-axis was position of gene set after ordering. Y -axis was score. Each line represents a gene in gene set. The length of lines indicates corresponding score.

The most significant four pathways in the GSEA-KEGG analysis were starch and sucrose metabolism (345 genes), flavonoid biosynthesis (73 genes), propanoate metabolism (50 genes), and fatty acid biosynthesis (41 genes) (Table S12, Fig. 10).

### Differentially expressed transcription factor genes

**Figure 11:**
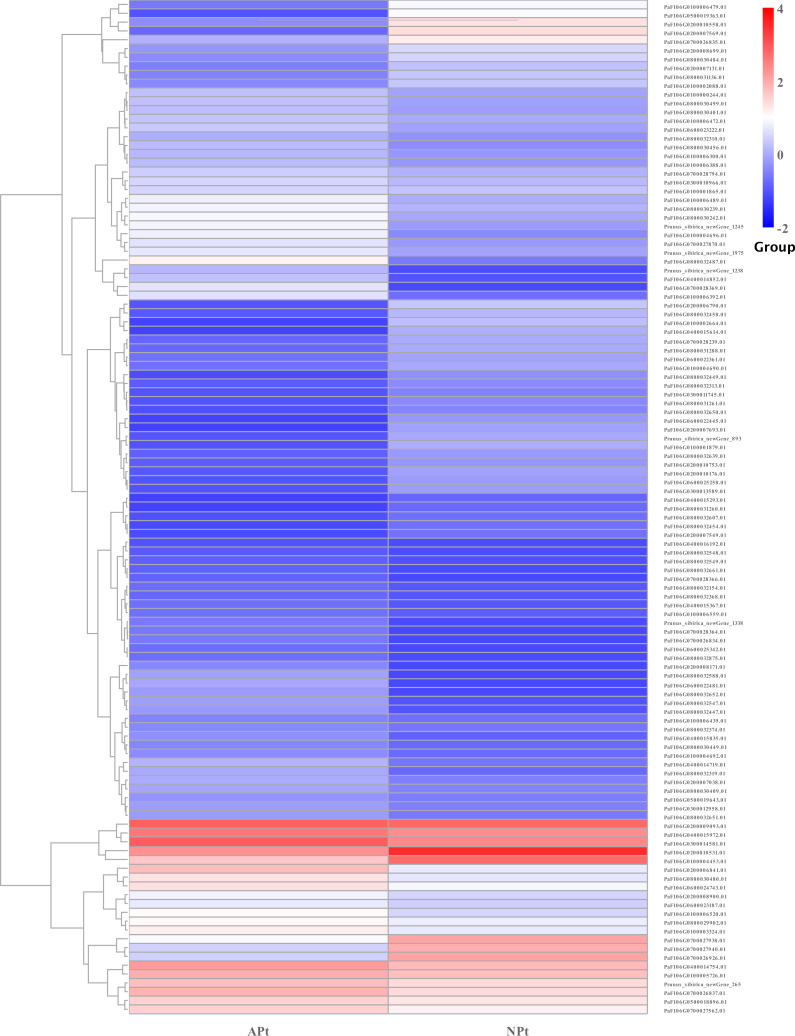
The relative expression pattern of NAC transcription factor among DEGs in *P*. *sibirica*.

**Figure 12:**
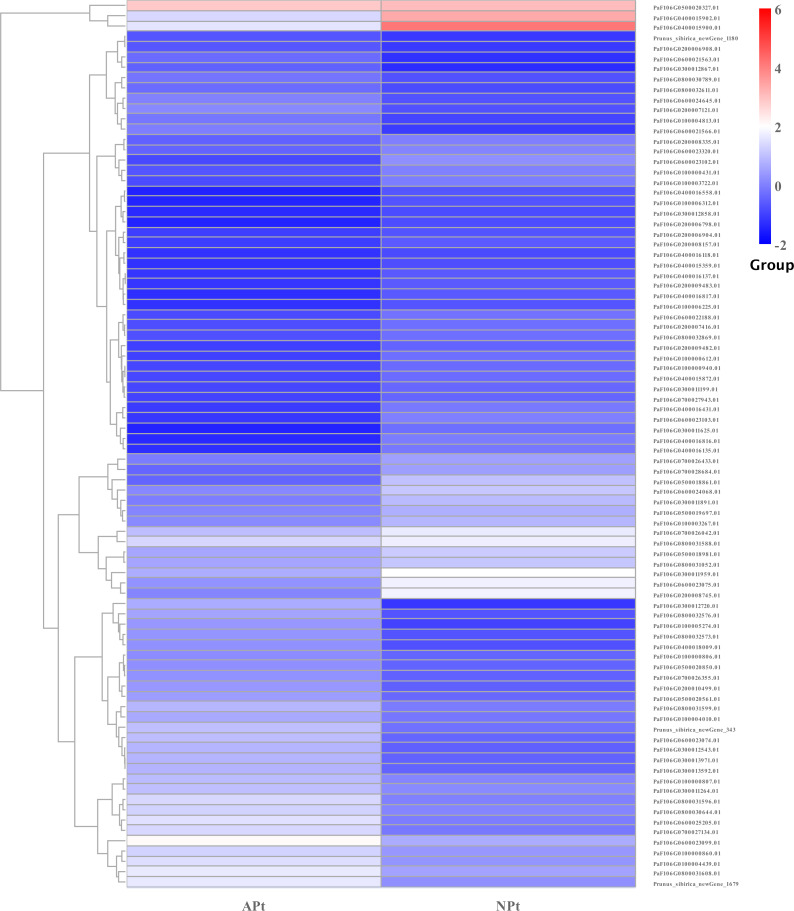
The relative expression pattern of bHLH transcription factor among DEGs in *P*. *sibirica*.

**Figure 13:**
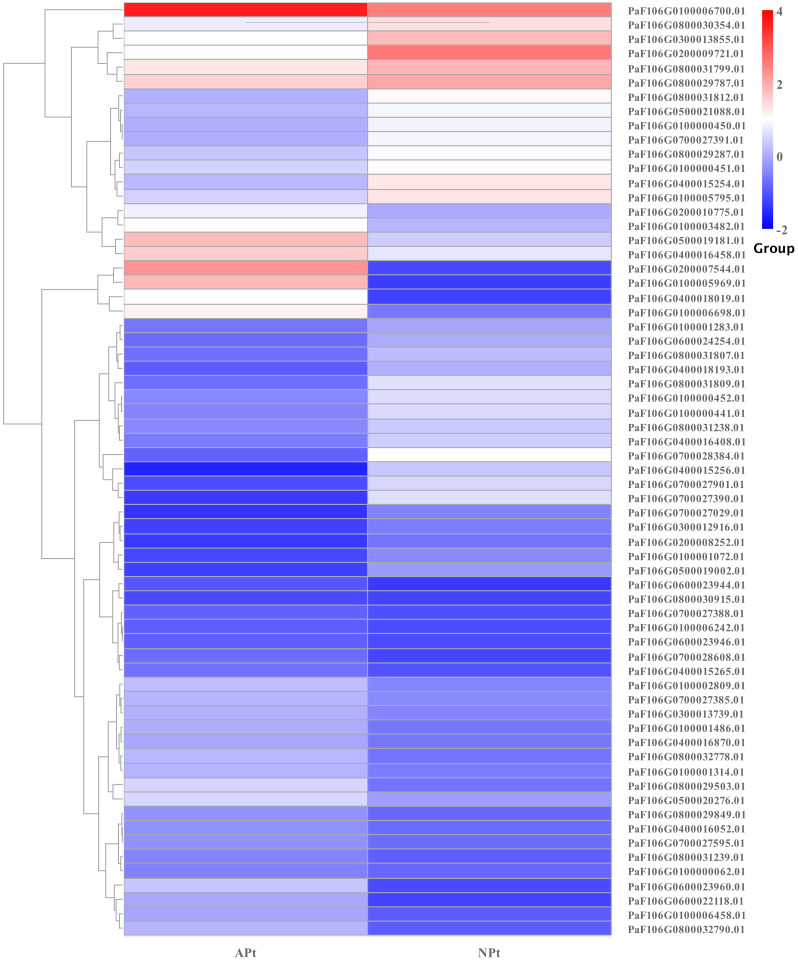
The relative expression pattern of B3 transcription factor among DEGs in *P*. *sibirica*.

**Figure 14:**
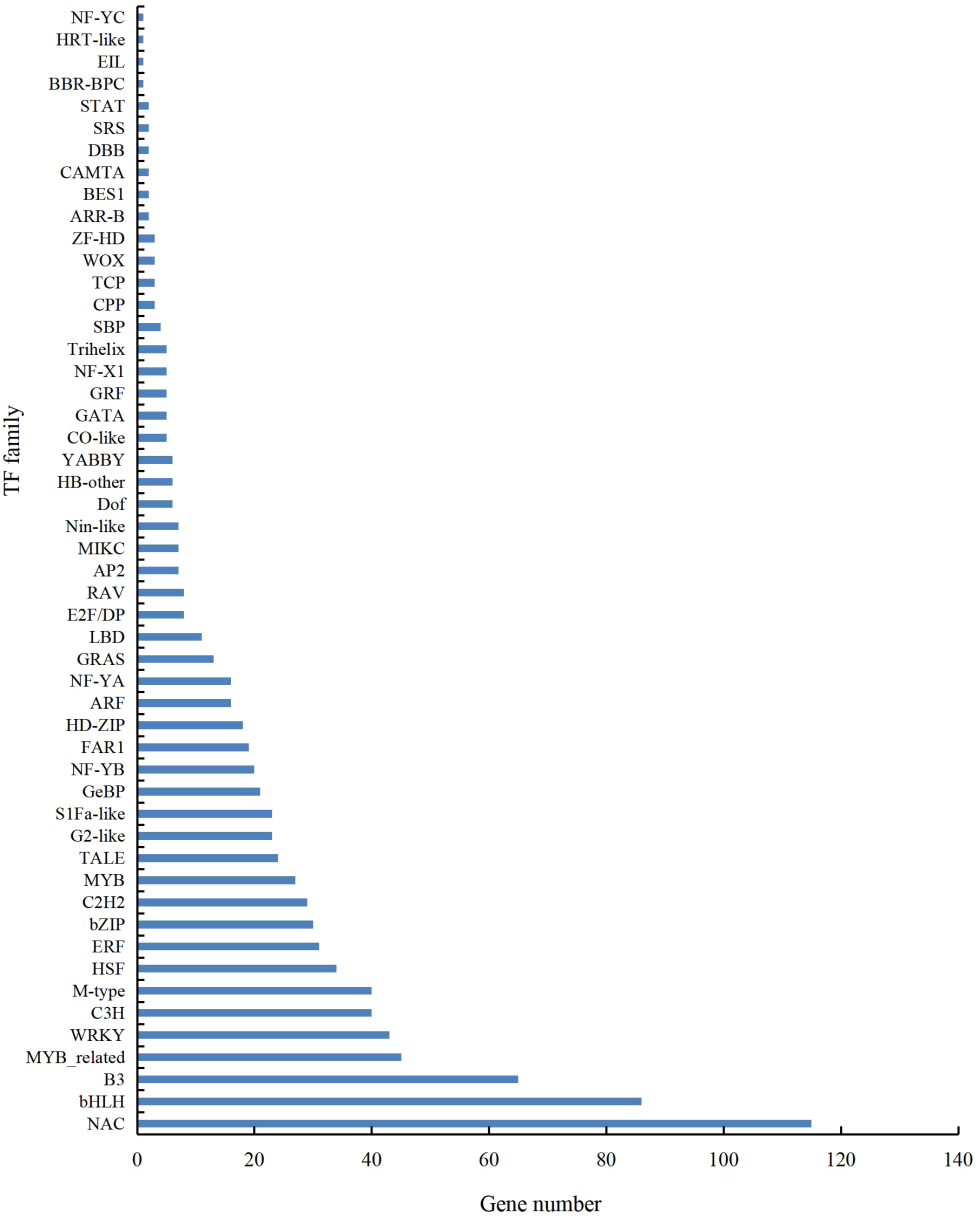
Distribution of transcription factor families in *P*. *sibirica*.

A total of 901 transcription factor (TF) genes were identified from all DEGs, which were divided into 51 TF families, including 434 downregulated DEGs and 467 upregulated DEGs (Table S13). NAC, bHLH and B3 contained the largest number of DEGs, and the gene expression pattern was shown in Figs. 11, 12 and 13. The NAC transcription factor family contained the largest number of DEGs, up to 115, of which 73 were upregulated and 42 were downregulated in flower buds with pistil abortion, respectively (Table S14). The bHLH transcription factor family contained 86 DEGs, of which 38 were upregulated and 48 were downregulated in flower buds with pistil abortion, respectively (Table S15). The B3 transcription factor family contained 65 DEGs, of which 34 were upregulated and 31 were downregulated in flower buds with pistil abortion, respectively (Table S16). Other transcription factor families contained more DEGs, MYB_Related contained 45 DEGs, WRKY contained 43 DEGs, C3H contained 40 DEGs, M-type contained 40 DEGs, HSF contained 34 DEGs, ERF contained 31 DEGs, bZIP contained 30 DEGs, C2H2 contained 29 DEGs, and MYB contained 27 DEGs (Fig. 14, Table S17).

### DEGs related to starch and sucrose metabolism

**Figure 15:**
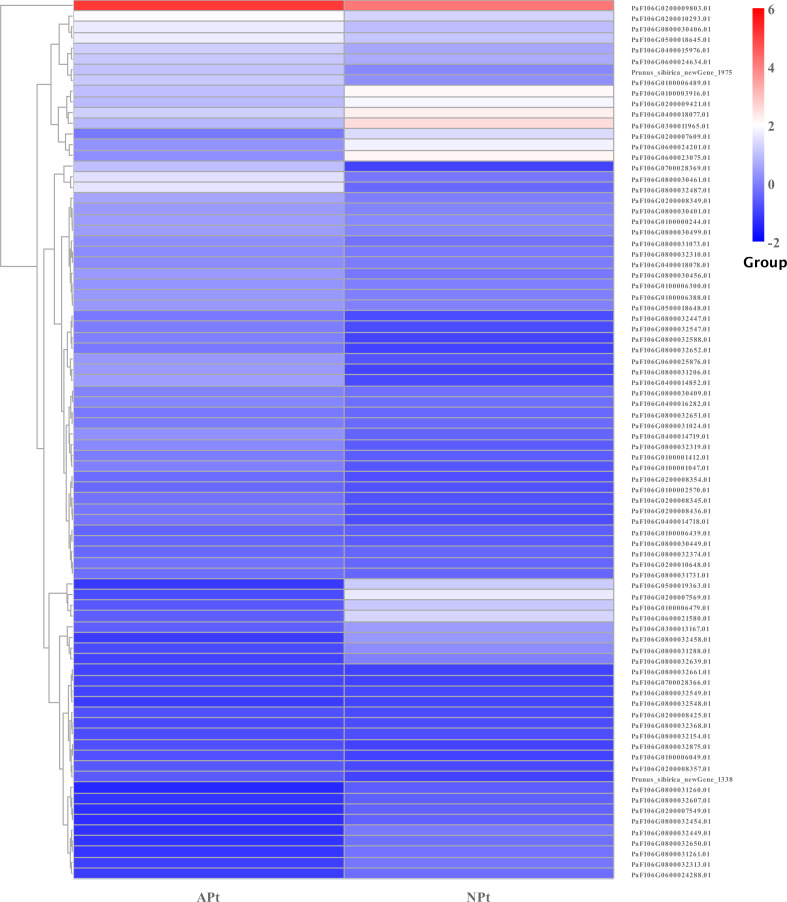
Cluster diagram of expression patterns of DEGs in *P*. *sibirica*.

In starch and sucrose metabolism, 82 related DEGs were identified, of which 58 were upregulated and 24 were downregulated, including 56 sucrose synthase, eight beta-glucosidase, three beta-fructofuranosidase, three glycogen phosphorylase, three trehalose 6-phosphate phosphatase, one alpha-amylase, one beta-amylase, one beta-carotene hydroxylase, one abscisate beta-glucosyltransferase, one endoglucanase, one xanthoxin dehydrogenase, one isoamylase, one abscisic acid 8′-hydroxylase, one sucrose-phosphate synthase. The expression patterns of these genes were shown in Fig. 15 (Table S18).

### DEGs related to phytohormone biosynthesis and signaling pathways

**Figure 16:**
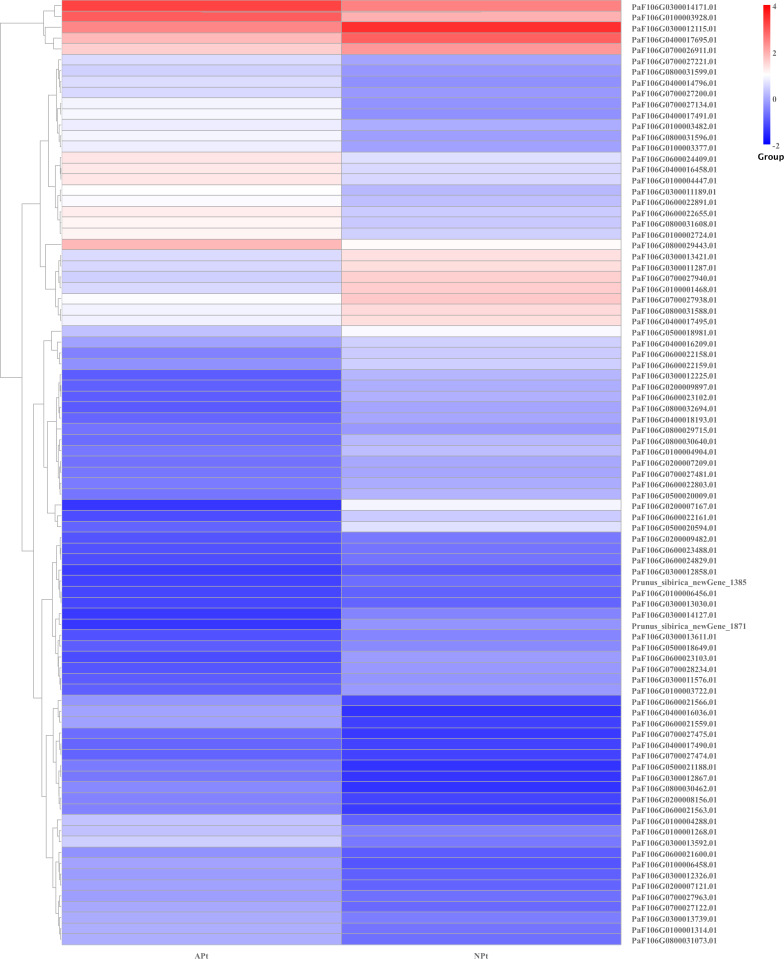
Cluster diagram of expression patterns of DEGs in *P*. *sibirica*.

There were 87 genes involved in the phytohormone biosynthesis and signaling pathways, of which 43 genes were upregulated and 44 genes were downregulated. There were 15 genes involved in the auxin signaling pathway, of which seven genes were upregulated and eight genes were downregulated. There were nine genes involved in cytokinin biosynthesis, of which three genes were upregulated and six genes were downregulated. There were two genes involved in the gibberellin signaling pathway, of which one gene was upregulated and one gene was downregulated. There were seven genes involved in the abscisic acid signaling pathway, of which four genes were upregulated and three genes were downregulated. There were four genes involved in the ethylene signaling pathway, of which two genes were upregulated and two genes were downregulated. There were 31 genes involved in the steroid signaling pathway, of which 15 genes were upregulated and 16 genes were downregulated. There were seven genes involved in the jasmonate signaling pathway, of which four genes were upregulated and three genes were downregulated. There were five genes involved in the salicylic acid signaling pathway, of which three genes were upregulated and two genes were downregulated. There were seven genes involved in the jasmonate acid signaling pathway, of which four genes were upregulated and three genes were downregulated (Table S19). The expression patterns of these genes were shown in Fig. 16.

### Real-time quantitative PCR (qRT-PCR) validation

**Figure 17:**
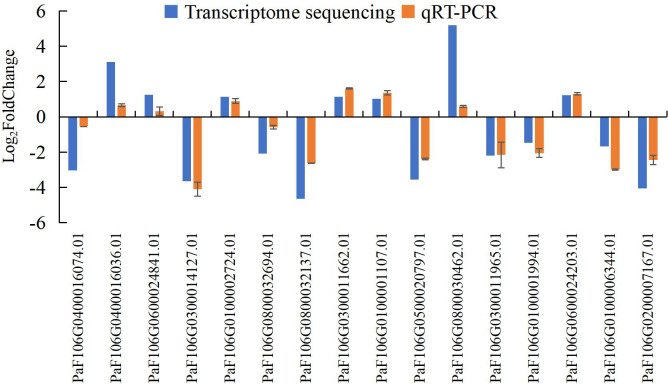
Comparison of transcriptome sequencing results and qRT-PCR analysis of gene expression levels in *P*. *sibirica*.

To confirm the accuracy and reproducibility of the transcriptome sequencing results, 16 representative genes were chosen to validate the expression levels between normal and abortive flowers by qRT-PCR. The relative expression of the selected genes was further compared with that of the transcriptome sequencing analysis. The relative trends in the expression patterns of the qRT-PCR results were all consistent with the transcriptome sequencing data, and the correlation coefficient was determined to be 0.797, supporting the reliability of the transcriptome sequencing results in this study (Fig. 17, Table S20, Table S21).

## Discussion

Gene expression and regulation play an important role in plant growth and development. Understanding the detailed information of genes is very important for understanding the molecular mechanism of the development process (Krizek, 2015). Transcriptome sequencing is an effective tool for DEG analysis during flower organ development and has been applied to *Annona squamosa* (Liu et al., 2017), *Punica granatum* (Chen et al., 2017a) and *Rhododendron simsii* (Liu et al., 2021). Normal pistil development directly determines the flowering and fruit setting phase in plants. Pistil abortion is also prevalent in *P. sibirica*, but its molecular mechanism is unclear. Therefore, this study analyzed its gene regulation mechanism by transcriptome sequencing analysis to identify DEGs and pathways related to pistil abortion. The sequence read was 150 bp with more than 92% high-quality reads, which met the requirements of sequencing library construction. Functional annotation of major databases showed that there were 30,543, 23,405, 23,357, 21,616, 18,941, 18,634, 13,897, and 8256 NR, GO, eggNOG, Pfam, KEGG, Swiss-Prot, KOG and COG annotations, respectively. Functional annotation analysis showed that the DEGs were related to a series of functions and different biological processes, such as flower development and cell process, indicating that pistil abortion may be controlled by multiple mechanisms of multiple genes. Transcriptome data provide an important reference for further study of the molecular mechanisms of pistil abortion or different biological processes.

Transcription factors are key regulatory proteins that mediate transcriptional regulation. They are widely present in plants and play an important role in reproductive development. In this study, we found 51 TF families, and showed different expression patterns in flower buds with normal and abortive pistils. In these TF families, bHLH, C2H2, MYB, GRF, TCP, and ZF-HD also regulate pistil development (Yuo et al., 2012; Nguyen et al., 2014; Hou et al., 2021). NAC, bHLH, MYB, WRKY and bZIP are highly expressed and have similar effects on the reproductive development of various plants (Sharma et al., 2012). These TFs may be involved in the pistil abortion of *P. sibirica*.

Carbohydrates, such as sucrose, glucose and starch, play an important role in signal transduction and energy in flower organ formation, flower induction of the flowering pathway, and reproductive development (Coneva et al., 2012). The starch content in flower buds of *Prunus armeniaca* var. *glabra* is the highest at the pistil differentiation stage, and the soluble sugar content in flower buds at different differentiation stages shows a trend of first decreased, then increased and then decreased (Zhao, 2020). The soluble sugar and soluble contents in complete flowers of *P. mume* varieties were higher than those in incomplete flowers, and the starch content was lower than that in incomplete flowers. The reason for pistil abortion in *P. mume* may be related to the catabolism of macromolecular nutrients in flower buds (Shi et al., 2011). Sucrose synthase (SS) is a key enzyme related to sucrose metabolism and carbohydrate composition (Chen et al., 2019). In our study, 56 DEGs related to sucrose synthase were identified in starch and sucrose metabolism, and they may be involved in the pistil abortion of *P. sibirica*.

Plant hormones play an important role in the growth and development of pistils (Marsch-Martínez & Folter, 2016; Zhao et al., 2018; Przedniczek, 2019). Auxins promote carpel initiation, pistil growth, and proper style and stigma formation (Marsch-Martínez & Folter, 2016). Pistil development is predominantly regulated by auxins (Chandler, 2011). The indoleacetic acid (IAA) content in normal flowers of *Prunus armeniaca* var. *glabra* was significantly higher than that in abortive flowers (Zhao, 2020). Exogenous auxin is beneficial for the generation of pistils (Przedniczek, 2019). Cytokinins play an important role in the development of the carpel margin meristem and its derived tissues, as well as in the formation of valve margin (Marsch-Martínez & Folter, 2016). Moreover, cytokinins regulate the number of ovules per pistil (Zuñiga Mayo et al., 2018). The cytokinin content in normal pistils of *P. mume* was significantly higher than that in abortive pistils (Shi et al., 2020). The contents of cytokinin and zirconium (ZR) in normal flowers of *Prunus armeniaca* var. *glabra* were significantly higher than those in abortive flowers (Zhao, 2020). Auxin-cytokinin interactions have also been shown to be necessary to control the development of meristem required for pistil development (Kurepa, Shull & Smalle, 2019). Gibberellic acid (GA) plays an important role in flower development, regulates plant gender differentiation, and inhibits pistil development at an appropriate level (Uno et al., 2000). Exogenous application of GA_3_ can partially prevent the pistil development of hermaphroditic *Jatropha curcas*, resulting in neutral flowers without stamens and pistils (Chen et al., 2017b). The content of GA in normal pistils of *P. mume* was significantly lower than that in abortive pistils (Shi et al., 2020), and the content of GA in normal flowers of *Prunus armeniaca* var. *glabra* was significantly lower than that in abortive flowers (Zhao, 2020). Abscisic acid (ABA) plays a crucial role in plant flowering induction (Duarte et al., 2019), flower bud differentiation (Yan et al., 2019), and flowering time (Emami, Kumar & Kempken, 2020). In the early stage of *Pharbitis nil* flower bud formation, low levels of endogenous ABA can promote flower bud formation (Wilmowicz et al., 2011). Exogenous application of ABA inhibited the emergence of *Phalaenopsis hybrida* flower buds, and a higher ABA dose had a stronger inhibitory effect (Wang et al., 2002). The ABA content of normal pistils of *P. mume* is significantly lower than that of abortive pistils (Shi et al., 2020), and the ABA content of normal flowers of *Prunus armeniaca* var. *glabra* is significantly lower than that of abortive flowers (Zhao, 2020). Ethylene has been verified as an upstream factor for pistil or ovule formation in many species, such as tobacco, pomegranate, and cucumber (Xin et al., 2019). A reduction in ethylene biosynthesis or perception delays the transition to pistillate flowering and reduces the number of pistillate flowers per plant in *Cucurbita sativus*, *C. melo*, and *C. pepo*, but has the opposite effect in *C. lanatus* (Manzano et al., 2011; Manzano et al., 2014; García et al., 2020). The ethylene content of fertile pistils of *P. mume* was significantly higher than that of abortive pistils (Shi et al., 2020). In our study, 87 genes were involved in the phytohormone biosynthesis and signaling pathways, and these genes may be involved in the pistil abortion of *P. sibirica*.

qRT-PCR is more advantageous than the classical reverse transcription-polymerase chain reaction in terms of quantitative accuracy, high sensitivity, and high throughput; thus, it has become the most commonly used method to detect and quantify the mRNA levels of target genes (Bustin et al., 2005). To verify the accuracy and reproducibility of transcriptome sequencing data, qRT-PCR was used to verify the expression levels of 16 randomly selected genes between normal and abortive flowers. The relative expression levels of the selected genes were compared with the transcriptome sequencing results. Although there were differences between the results of qRT-PCR and the gene expression levels obtained by transcriptome sequencing, the expression patterns, and trends of genes were consistent. This shows that the accuracy of transcriptome sequencing results is high and can be used to further analyze the dynamic changes in genes.

Based on transcriptome sequencing technology and bioinformatics analysis, we explored the key regulatory genes and metabolic pathways related to pistil abortion in *P. sibirica*. We can verify the function of the key genes in further research, and overcome pistil abortion in production practice through transgenic technology, so as to improve fruit yield and quality.

## Conclusions

In this study, transcriptome sequencing was used to analyze the regulation and expression patterns of genes in *P. sibirica* flower buds with normal and abortive pistils, and a total of 1,950 DEGs were identified. Pathways such as plant-pathogen interaction, starch and sucrose metabolism, and plant hormone signal transduction contained the largest number of DEGs. The NAC, bHLH, and B3 transcription factor families contained the largest number of DEGs. By analyzing the gene expression patterns and trends related to plant hormone biosynthesis and signaling pathways, we found that hormones such as auxin, cytokinin, gibberellin, abscisic acid, and ethylene play important roles in the process of pistil abortion, and related genes are involved in hormone synthesis and expression, regulation of hormone content, and promotion of abortion. qRT-PCR verified that the gene expression levels were consistent with the transcriptome sequencing results. This study provides a theoretical basis and scientific basis for further research on the molecular mechanisms of *P. sibirica* pistil abortion.

##  Supplemental Information

10.7717/peerj.14366/supp-1 Table S1 The power analysis calculation

10.7717/peerj.14366/supp-2 Table S2 The primers used for qRT-PCR

10.7717/peerj.14366/supp-3 Table S3 The results of the total RNA quality

10.7717/peerj.14366/supp-4 Table S4 Summary of transcriptome sequencing data GC content: Percentage of G, C in clean data. ≥Q30%: Percentage of bases with Q-score no less than Q30.

10.7717/peerj.14366/supp-5 Table S5 The qualities of transcriptome sequencing and sequence alignment results

10.7717/peerj.14366/supp-6 Table S6 Unigene information annotated in different databases

10.7717/peerj.14366/supp-7 Table S7 Detailed information of DEGs in GO enrichment analysis

10.7717/peerj.14366/supp-8 Table S8 Detailed information of DEGs in KEGG pathway enrichment analysis

10.7717/peerj.14366/supp-9 Table S9 KEGG pathway enrichment analysis for DEGs

10.7717/peerj.14366/supp-10 Table S10 Detailed information of DEGs in COG classification

10.7717/peerj.14366/supp-11 Table S11 GSEA analysis based on GO functional classification in *P. sibirica*

10.7717/peerj.14366/supp-12 Table S12 GSEA analysis based on KEGG pathway in *P. sibirica*

10.7717/peerj.14366/supp-13 Table S13 Differentially expressed transcription factor genes

10.7717/peerj.14366/supp-14 Table S14 The DEGs in NAC transcription factor family

10.7717/peerj.14366/supp-15 Table S15 The DEGs in bHLH transcription factor family

10.7717/peerj.14366/supp-16 Table S16 The DEGs in B3 transcription factor family

10.7717/peerj.14366/supp-17 Table S17 Distribution of transcription factor families

10.7717/peerj.14366/supp-18 Table S18 DEGs related to starch and sucrose metabolism

10.7717/peerj.14366/supp-19 Table S19 DEGs related to phytohormone biosynthesis and signaling pathways

10.7717/peerj.14366/supp-20 Table S20 Raw data of Ct value

10.7717/peerj.14366/supp-21 Table S21 Detailed information of DEGs in qRT-PCR

## Additional Information and Declarations

### Data Availability

The following information was supplied regarding data availability: The data is available at NCBI GEO: GSE192952 and the raw data are in the Supplemental Tables.

## References

[ref-1] Bustin SA, Benes V, Nolan T, Pfaffl MW (2005). Quantitative real-time RT-PCR—a perspective. Journal of Molecular Endocrinology.

[ref-2] Chandler JW (2011). The hormonal regulation of flower development. Journal of Plant Growth Regulation.

[ref-3] Chen CL, Zhao T, Guo R, Liu SH, Zhang JQ (2021). Investigation of biological characteristics and observation of pistil abortion during flower bud development of *Prunus armeniaca* L. var. glabra Sun S. X. Acta Botanica Boreali-Occidentalia Sinica.

[ref-4] Chen LN, Zhang J, Li HX, Niu J, Xue H, Liu BB, Wang Q, Luo X, Zhang FH, Zhao DG, Cao SY (2017b). Transcriptomic analysis reveals candidate genes for female sterility in pomegranate flowers. Frontiers in Plant Science.

[ref-5] Chen MS, Pan BZ, Fu QT, Tao YB, Martínez-Herrera J, Niu LJ, j Ni, Dong YL, Zhao M-L, Xu Z-F (2017a). Comparative transcriptome analysis between gynoecious and monoecious plants identifies regulatory networks controlling sex determination in *Jatropha curcas*. Frontiers in Plant Science.

[ref-6] Chen X-L, Wang L-C, Li T, Yang QC, Guo W-Z (2019). Sugar accumulation and growth of lettuce exposed to different lighting modes of red and blue LED light. Scientific Reports.

[ref-7] Coneva V, Guevara D, Rothstein SJ, Colasanti J (2012). Transcript and metabolite signature of maize source leaves suggests a link between transitory starch to sucrose balance and the autonomous floral transition. Journal of Experimental Botany.

[ref-8] Duarte KE, De Souza WR, Santiago TR, Sampaio BL, Ribeiro AP, Cotta MG, Da Cunha BADB, Marraccini PRR, Kobayashi AK, Molinari HBC (2019). Identification and characterization of core abscisic acid (ABA) signaling components and their gene expression profile in response to abiotic stresses in *Setaria viridis*. Scientific Reports.

[ref-9] Emami H, Kumar A, Kempken F (2020). Transcriptomic analysis of poco1, a mitochondrial pentatricopeptide repeat protein mutant in *Arabidopsis thaliana*. BMC Plant Biology.

[ref-10] Florea L, Song L, Salzberg SL (2013). Thousands of exon skipping events differentiate among splicing patterns in sixteen human tissues. F1000Research.

[ref-11] García A, Aguado E, Martínez C, Loska D, Beltrán S, Valenzuela JL, Garrido D, Jamilena M (2020). The ethylene receptors CpETR1A and CpETR2B cooperate in the control of sex determination in *Cucurbita pepo*. Journal of Experimental Botany.

[ref-12] Grundy MML, Lapsley K, Ellis PR (2016). A review of the impact of processing on nutrient bioaccessibility and digestion of almonds. International Journal of Food Science and Technology.

[ref-13] Hou C, Tian YX, Wang YL, Lian HM, Liang DC, Shi SQ, Deng N, He B (2021). Revealing the developmental dynamics in male strobilus transcriptome of Gnetum luofuense using nanopore sequencing technology. Scientific Reports.

[ref-14] Jin L (2018). Exploration of SSR loci associated with mainly economic traits and analysis of expression in *Armeniaca sibirica*. Master’s Thesis.

[ref-15] Khan A, Fornés Oriol, Stigliani A, Gheorghe M, Castro-Mondragon JA (2017). JASPAR 2018: update of the open-access database of transcription factor binding profiles and its web framework. Nucleic Acids Research.

[ref-16] Kim D, Langmead B, Salzberg SL (2015). HISAT: a fast spliced aligner with low memory requirements. Nature Methods.

[ref-17] Krizek BA (2015). AINTEGUMENTA-LIKE genes have partly overlapping functions with AINTEGUMENTA but make distinct contributions to *Arabidopsis thaliana* flower development. Journal of Experimental Botany.

[ref-18] Kurepa J, Shull TE, Smalle JA (2019). Antagonistic activity of auxin and cytokinin in shoot and root organs. Plant Direct.

[ref-19] Li LH, Ma FW (2001). Morphological studies on flower bud differentiation in different apricot varieties. Journal of Northwest A&F University (Social Science Edition).

[ref-20] Li QF, Zhang L, Pan FF, Guo WL, Chen BH, Yang HL, Wang GY, Li XZ (2020). Transcriptomic analysis reveals ethylene signal transduction genes involved in pistil development of pumpkin. PeerJ.

[ref-21] Liu KD, Li HL, Li WJ, Zhong JD, Chen Y, Shen CJ, Yuan CC (2017). Comparative transcriptomic analyses of normal and malformed flowers in sugar apple (*Annona squamosa* L.) to identify the differential expressed genes between normal and malformed flowers. BMC Plant Biology.

[ref-22] Liu MY, Ma ZT, Zheng TR, Sun WJ, Zhang YJ, Jin WQ, Zhan JY, Cai YT, Tang YJ, Wu Q, Tang ZZ, Bu TL, Li CL, Chen H (2018). Insights into the correlation between Physiological changes in and seed development of tartary buckwheat (*Fagopyrum tataricum* Gaertn.). BMC Genomics.

[ref-23] Liu QL, Liaquat F, He YF, Munis MFH, Zhang CY (2021). Functional annotation of a full-Length transcriptome and identification of genes associated with flower development in *Rhododendron simsii* (Ericaceae). Plants-Basel.

[ref-24] Mai YT, Huo K, Yu HY, Zhou N, Shui LY, Liu Y, Zhang CX, Niu J, Wang LB (2020). Using lipidomics to reveal details of lipid accumulation in developing Siberian apricot (*Prunus sibirica* L.) seed kernels. Global Change Biology Bioenergy.

[ref-25] Manzano S, Martínez C, García JM, Megías Z, Jamilena M (2014). Involvement of ethylene in sex expression and female flower development in watermelon (*Citrullus lanatus*). Plant Physiology and Biochemistry.

[ref-26] Manzano S, Martínez C, Megías Z, Gómez P, Garrido D, Jamilena M (2011). The role of ethylene and brassinosteroids in the control of sex expression and flower development in *Cucurbita pepo*. Plant Growth Regulation.

[ref-27] Mao XZ, Cai T, Olyarchuk JG, Wei LP (2005). Automated genome annotation and pathway identification using the KEGG Orthology (KO) as a controlled vocabulary. Bioinformatics.

[ref-28] Marsch-Martínez N, Folter SD (2016). Hormonal control of the development of the gynoecium. Current Opinion in Plant Biology.

[ref-29] Nguyen CV, Vrebalov JT, Gapper NE, Zheng Y, Zhong S, Fei ZJ, Giovannoni JJ (2014). Tomato GOLDEN2-LIKE transcription factors reveal molecular gradients that function during fruit development and ripening. Plant Cell.

[ref-30] Niu J, Zhu BQ, Cai J, Li PX, Wang LB, Dai HT, Qiu L, Yu HY, Ha DL, Zhao HY, Zhang ZX, Lin SZ (2014). Selection of reference genes for gene expression studies in Siberian Apricot (*Prunus sibirica* L.) germplasm using quantitative real-time PCR. PLOS ONE.

[ref-31] Przedniczek M (2019). Comprehensive insight into Gibberellin- and Jasmonate-mediated stamen development. Genes.

[ref-32] Robinson MD, McCarthy DJ, Smyth GK (2010). edgeR: a bioconductor package for differential expression analysis of digital gene expression data. Bioinformatics.

[ref-33] Rosatia A, Caporali S, Paolettia A, Famiani F (2011). Pistil abortion is related to ovary mass in olive (*Olea europaea* L.). Scientia Horticulturae.

[ref-34] Sharma R, Agarwal P, Ray S, Deveshwar P, Sharma P, Sharma N, Nijhawan A, Jain M, Singh AK, Singh VP, Khurana JP, Tyagi AK, Kapoor S (2012). Expression dynamics of metabolic and regulatory components across stages of panicle and seed development in indica rice. Functional & Integrative Genomics.

[ref-35] Shen HX, Kong Y, Yao YC, Zhang R, Fu ZF (2007). Research progress on pistil abortion of apricot flower. China Fruits.

[ref-36] Shi T, Iqbal S, Ayaz A, Bai Y, Pan ZP, Ni XP, Hayat F, Bilal MS, Razzaq MK, Gao ZH (2020). Analyzing differentially expressed genes and pathways associated with pistil abortion in Japanese apricot via RNA-Seq. Genes.

[ref-37] Shi T, Zhang QL, Gao ZH, Zhang Z, Zhuang WB (2011). Analyses on pistil differentiation process and related biochemical indexes of two cultivars of *Prunus mume*. Journal of Plant Resources and Environment.

[ref-38] Sun HL (2014). Isolation and functional analysis of pistil development related gene in Japanese apricot. Master’s Thesis.

[ref-39] Uno Y, Furihata T, Abe H, Yoshida R, Shinozaki K, Shinozaki Y (2000). Arabidopsis basic leucine zipper transcription factors involved in an abscisic acid-dependent signal transduction pathway under drought and high-salinity conditions. Proceedings of the National Academy of Sciences of the United States of America.

[ref-40] Wang B, Ding G, Tong D, Liu S (2000). A Study on the pistils abortion of the kernel apricot. Journal of Shanxi Agricultural Sciences.

[ref-41] Wang LB, Yu HY (2012). Biodiesel from Siberian apricot (*Prunus sibirica* L.) seed kernel oil. Bioresource Technology.

[ref-42] Wang WY, Chen WS, Chen WH, Hung LS, Chang PS (2002). Influence of abscisic acid on flowering in *Phalaenopsis hybrida*. Proceedings of the National Academy of Sciences of the United States of America.

[ref-43] Wang Z, Liu HB, Liu J, Li YY, Wu RL, Pang XM (2014). Mining new microsatellite markers for Siberian apricot (*Prunus sibirica* L.) from SSR-enriched genomic library. Scientia Horticulturae.

[ref-44] Wetzstein HY, Ravid N, Wilkins E, Martinelli AP (2011). A morphological and histological characterization of bisexual and male flower types in pomegranate. Journal of the American Society for Horticultural Science.

[ref-45] Wilmowicz E, Frankowski K, Glazińska P, Kesy J, Kopcewicz J (2011). Involvement of ABA in flower induction of *Pharbitis nil*. Acta Societatis Botanicorum Poloniae.

[ref-46] Xin GL, Liu JQ, Liu J, Ren XL, Du XM (2019). Anatomy and RNA-Seq reveal important gene pathways regulating sex differentiation in a functionally Androdioecious tree, Tapiscia sinensis. BMC Plant Biology.

[ref-47] Yan BB, Hou JL, Cui J, He C, Li WB (2019). The effects of endogenous hormones on the flowering and fruiting of *Glycyrrhiza uralensis*. Plants-Basel.

[ref-48] Yin MY, Zhu XC, Liu HM, Liu JQ, Wuyun TN (2018). Flower phenotypic variations of germplasm resources of Siberian apricot (*Armeniaca sibirica*). Journal of Northwest A&F University(Social Science Edition).

[ref-49] You T, Yamashita Y, Kanamori H, Matsumoto T, Lundqvist U, Sato K, Ichii M, Jobling SA, Taketa S (2012). A SHORT INTERNODES (SHI) family transcription factor gene regulates awn elongation and pistil morphology in barley. Journal of Experimental Botany.

[ref-50] Young MD, Wakefield MJ, Smyth GK, Oshlack A (2010). Gene ontology analysis for RNA-seq: accounting for selection bias. Genome Biology.

[ref-51] Zhang DX, Hu LZ, Xiao WT, Ni XL, Hu JQ (2013). Morphological and anatomical study on female sterility in *Camptotheca acuminata* decne. Bulletin of Botanical Research.

[ref-52] Zhang L (2017). Identification of miRNAs involved in the development and differentiation of fertile and sterile flowers in *Viburnum macrocephalum* f. keteleeri. Master’s Thesis.

[ref-53] Zhang J (2022). Study of pistil abortion in *Prunus sibirica* based on physiological and biochemical characteristics. Master’s Thesis.

[ref-54] Zhao T (2020). Observation of flower bud differentiation and transcriptome analysis of pistil abortion in ‘Li guang xing’. Master’s Thesis.

[ref-55] Zhao Y, Zhang YZ, Wang LJ, Wang XR, Xu W, Gao XY, Liu BS (2018). Mapping and Functional Analysis of a Maize Silkless Mutant *sk-A7110*. Frontiers in Plant Science.

[ref-56] Zuñiga Mayo VM, Baños Bayardo CR, Díaz-Ramírez D, Marsch-Martínez N, Folter SD (2018). Conserved and novel responses to cytokinin treatments during flower and fruit development in *Brassica napus* and *Arabidopsis thaliana*. Scientific Reports.

