# Supplementary material for: Mining for genes related to pistil abortion in Prunus sibirica L
Source: PeerJ. 2022 Nov 15;10:e14366. doi: 10.7717/peerj.14366 (PMC9673769; doi:10.7717/peerj.14366)
Supplement: Table S2 [file peerj-10-14366-s002.docx]

**Table S2 The primers used for qRT-PCR**

| **Gene ID** | **Primer** | **Primer sequence (5’-3’)** | **Tm** |
| --- | --- | --- | --- |
| PaF106G0400016074.01 | PaF106G0400016074.01-F | GGAAAGAGAAGGTGAACAAGGAT | 58.39 |
|  | PaF106G0400016074.01-R | CTCAACAAAATCAGCACCACAC | 58.21 |
| PaF106G0100002724.01 | PaF106G0100002724.01-F | GGTGATGCGTAAAAGAACAAATG | 56.60 |
|  | PaF106G0100002724.01-R | GCAGTCCTAAGCCAGAAACAGAAT | 60.26 |
| PaF106G0200007167.01 | PaF106G0200007167.01-F | GCAACCCACCTATTCATGTTTAC | 58.39 |
|  | PaF106G0200007167.01-R | GATCCTAGAAGAGTCCTCAGTCTCA | 61.98 |
| PaF106G0100006344.01 | PaF106G0100006344.01-F | CACTTTGGCTTCTTTGGGATT | 56.06 |
|  | PaF106G0100006344.01-R | TTGACGGTTTGTGGTGTTCTC | 58.01 |
| PaF106G0400016036.01 | PaF106G0400016036.01-F | CCACCATTCTCTCCCTCATCA | 59.97 |
|  | PaF106G0400016036.01-R | ACGACCACATTGCCCACAGT | 59.85 |
| PaF106G0300011662.01 | PaF106G0300011662.01-F | GCCCACAACTCATCCACCCT | 61.90 |
|  | PaF106G0300011662.01-R | CACCATCTTCCATCCCTCCTC | 61.92 |
| PaF106G0100001107.01 | PaF106G0100001107.01-F | CGAAAGTTCCAGAAGACCATCC | 60.07 |
|  | PaF106G0100001107.01-R | GACATCACCAACCAATCACTGC | 60.07 |
| PaF106G0800032694.01 | PaF106G0800032694.01-F | TCTCAATCAACACGCCTCACC | 59.97 |
|  | PaF106G0800032694.01-R | ACTCCCACACCATCCTCACG | 61.90 |
| PaF106G0600024203.01 | PaF106G0600024203.01-F | GTGTCCCTTGCGGTTTCTTAC | 59.97 |
|  | PaF106G0600024203.01-R | TCATCAAGCTGCTGACCAATT | 56.06 |
| PaF106G0600024841.01 | PaF106G0600024841.01-F | GGGATTCCATTTCCTACACTCTC | 60.17 |
|  | PaF106G0600024841.01-R | GGAGCCAATGAACCCTCTACTT | 60.07 |
| PaF106G0300011965.01 | PaF106G0300011965.01-F | GGAACCCAGAATACCTCACTCTAG | 61.97 |
|  | PaF106G0300011965.01-R | CAAGCCCCACTTCAATGTCTATA | 58.39 |
| PaF106G0100001994.01 | PaF106G0100001994.01-F | TTTGGGGGTTCCTCTGTGTG | 59.85 |
|  | PaF106G0100001994.01-R | AATCAGCAGGGTTTCTGTTAATG | 56.60 |
| PaF106G0500020797.01 | PaF106G0500020797.01-F | TTTGAAAAGATGCGGGAAGA | 53.70 |
|  | PaF106G0500020797.01-R | TATAGCCGACCACCTGTTGC | 59.85 |
| PaF106G0300014127.01 | PaF106G0300014127.01-F | CTATTTTGCTATCTATCGCCGTT | 56.00 |
|  | PaF106G0300014127.01-R | AAGTTGGATTTCTGCCATACCTT | 56.00 |
| PaF106G0800030462.01 | PaF106G0800030462.01-F | AAACAGGGACAGAAGCCGAT | 57.80 |
|  | PaF106G0800030462.01-R | CCCCAAATACCTACATAGCAATC | 58.39 |
| PaF106G0800032137.01 | PaF106G0800032137.01-F | TGACTCCCCAATGCTTGACTG | 59.97 |
|  | PaF106G0800032137.01-R | GGCCACCTAACTGATTTCTACTG | 60.17 |
| 18SrRNA | 18SrRNA-F | AAACGGCTACCACATCCA | 55.02 |
| 18SrRNA | 18SrRNA-R | CACCAGACTTGCCCTCCA | 59.58 |
