# Supplementary material for: Mining for genes related to pistil abortion in Prunus sibirica L
Source: PeerJ. 2022 Nov 15;10:e14366. doi: 10.7717/peerj.14366 (PMC9673769; doi:10.7717/peerj.14366)
Supplement: Table S3 [file peerj-10-14366-s003.docx]

**Table S3 The results of the total RNA quality**

| **Samples** | **Concentration (ng/ul)** | **Total (ug)** | **OD260/280** | **OD260/230** | **RIN** | **28S/18S** |
| --- | --- | --- | --- | --- | --- | --- |
| APt1 | 925.2 | 21.28 | 2.16 | 2.21 | 9.0 | 1.97 |
| APt2 | 716.6 | 16.48 | 2.11 | 1.84 | 8.9 | 2.09 |
| APt3 | 718.2 | 16.52 | 2.10 | 1.95 | 8.9 | 2.06 |
| NPt1 | 615.3 | 14.15 | 2.14 | 1.80 | 8.7 | 1.94 |
| NPt2 | 331.5 | 7.62 | 2.18 | 1.16 | 9.2 | 2.04 |
| NPt3 | 768.2 | 17.67 | 2.11 | 1.66 | 9.0 | 2.05 |
