# Supplementary material for: Mining for genes related to pistil abortion in Prunus sibirica L
Source: PeerJ. 2022 Nov 15;10:e14366. doi: 10.7717/peerj.14366 (PMC9673769; doi:10.7717/peerj.14366)
Supplement: Table S4 — GC content: Percentage of G, C in clean data. ≥Q30%: Percentage of bases with Q-score no less than Q30. [file peerj-10-14366-s004.docx]

**Table S4 Summary of transcriptome sequencing data**

| **Samples** | **Clean reads** | **Clean bases** | **GC Content (%)** | **Q30 value (%)** |
| --- | --- | --- | --- | --- |
| APt1 | 24,772,993 | 7,409,911,834 | 45.95 | 92.63 |
| APt2 | 23,130,820 | 6,916,694,026 | 45.94 | 92.92 |
| APt3 | 26,872,112 | 8,043,438,076 | 45.89 | 92.79 |
| NPt1 | 27,048,846 | 8,096,571,394 | 45.91 | 92.01 |
| NPt2 | 22,462,256 | 6,713,330,540 | 45.89 | 92.30 |
| NPt3 | 26,016,684 | 7,784,268,846 | 46.03 | 92.89 |

GC content: Percentage of G, C in clean data.

≥Q30%: Percentage of bases with Q-score no less than Q30.
