# Supplementary material for: Mining for genes related to pistil abortion in Prunus sibirica L
Source: PeerJ. 2022 Nov 15;10:e14366. doi: 10.7717/peerj.14366 (PMC9673769; doi:10.7717/peerj.14366)
Supplement: Table S5 [file peerj-10-14366-s005.docx]

**Table S5 The qualities of transcriptome sequencing and sequence alignment results**

| **Samples** | **Total Reads** | **Mapped Reads** | **Uniq Mapped Reads** | **Multiple Map Reads** | **Reads Map to '+'** | **Reads Map to '-'** |
| --- | --- | --- | --- | --- | --- | --- |
| APt1 | 49,545,986 | 46,013,156 (92.87%) | 44,322,228 (89.46%) | 1,690,928 (3.41%) | 22,879,957 (46.18%) | 22,937,712 (46.30%) |
| APt2 | 46,261,640 | 42,989,119 (92.93%) | 41,417,638 (89.53%) | 1,571,481 (3.40%) | 21,390,103 (46.24%) | 21,423,891 (46.31%) |
| APt3 | 53,744,224 | 50,005,754 (93.04%) | 48,261,447 (89.80%) | 1,744,307 (3.25%) | 24,880,778 (46.29%) | 24,927,602 (46.38%) |
| NPt1 | 54,097,692 | 49,975,874 (92.38%) | 48,212,608 (89.12%) | 1,763,266 (3.26%) | 24,870,001 (45.97%) | 24,908,707 (46.04%) |
| NPt2 | 44,924,512 | 41,836,069 (93.13%) | 40,432,750 (90.00%) | 1,403,319 (3.12%) | 20,822,302 (46.35%) | 20,864,766 (46.44%) |
| NPt3 | 52,033,368 | 48,408,232 (93.03%) | 46,757,807 (89.86%) | 1,650,425 (3.17%) | 24,091,635 (46.30%) | 24,134,716 (46.38%) |
