# Supplementary material for: Mining for genes related to pistil abortion in Prunus sibirica L
Source: PeerJ. 2022 Nov 15;10:e14366. doi: 10.7717/peerj.14366 (PMC9673769; doi:10.7717/peerj.14366)
Supplement: Table S6 [file peerj-10-14366-s006.docx]

**Table S6 Unigene information annotated in different databases**

| **Database** | **Numbers of Unigenes** | **Percentage(%)** |
| --- | --- | --- |
| NR | 30543 | 89.7 |
| GO | 23405 | 68.7 |
| eggNOG | 23357 | 68.6 |
| Pfam | 21616 | 63.5 |
| KEGG | 18941 | 55.6 |
| Swiss-Prot | 18634 | 54.7 |
| KOG | 13897 | 40.8 |
| COG | 8256 | 24.2 |
| Total Unigenes | 30627 | 89.9 |
