# Supplementary material for: Mining for genes related to pistil abortion in Prunus sibirica L
Source: PeerJ. 2022 Nov 15;10:e14366. doi: 10.7717/peerj.14366 (PMC9673769; doi:10.7717/peerj.14366)
Supplement: Table S9 [file peerj-10-14366-s009.docx]

**Table S9 KEGG pathway enrichment analysis for DEGs**

| **ID** | **Pathway** | **DEGs** |
| --- | --- | --- |
| ko04626 | Plant-pathogen interaction | 104 |
| ko00500 | Starch and sucrose metabolism | 78 |
| ko04075 | Plant hormone signal transduction | 51 |
| ko00940 | Phenylpropanoid biosynthesis | 40 |
| ko04016 | MAPK signaling pathway - plant | 38 |
| ko03040 | Spliceosome | 34 |
| ko00040 | Pentose and glucuronate interconversions | 31 |
| ko00941 | Flavonoid biosynthesis | 24 |
| ko04141 | Protein processing in endoplasmic reticulum | 22 |
| ko04144 | Endocytosis | 22 |
| ko00945 | Stilbenoid, diarylheptanoid and gingerol biosynthesis | 20 |
| ko00520 | Amino sugar and nucleotide sugar metabolism | 20 |
| ko00460 | Cyanoamino acid metabolism | 18 |
| ko03008 | Ribosome biogenesis in eukaryotes | 15 |
| ko00592 | alpha-Linolenic acid metabolism | 15 |
| ko00052 | Galactose metabolism | 15 |
| ko04712 | Circadian rhythm-plant | 14 |
| ko02010 | ABC transporters | 14 |
| ko03010 | Ribosome | 13 |
| ko03440 | Homologous recombination | 13 |
| ko00053 | Ascorbate and aldarate metabolism | 12 |
| ko03013 | RNA transport | 12 |
| ko01200 | Carbon metabolism | 12 |
| ko00270 | Cysteine and methionine metabolism | 11 |
| ko00564 | Glycerophospholipid metabolism | 11 |
| ko01230 | Biosynthesis of amino acids | 11 |
| ko04120 | Ubiquitin mediated proteolysis | 11 |
| ko00350 | Tyrosine metabolism | 11 |
| ko00950 | Isoquinoline alkaloid biosynthesis | 10 |
| ko00902 | Monoterpenoid biosynthesis | 10 |
